# Adapting the MPOWER policy framework for fossil fuels and public health: reflections on content and process

**DOI:** 10.1177/17579139251342161

**Published:** 2025-06-28

**Authors:** T Keller, I Braithwaite, A Brook, J Beagley, S Narayan, TA Deivanayagam

**Affiliations:** UK Faculty of Public Health, London, UK; UK Faculty of Public Health, Primary Care and Public Health Research Department, University College London, London, UK; UK Faculty of Public Health, London, UK; Global Climate and Health Alliance, Richmond, CA, USA; Global Climate and Health Alliance, Richmond, CA, USA; UK Faculty of Public Health, Institute for Global Health, University College London, 30 Guilford Street, London, WC1N 1EH, UK

## Abstract

We argue in the paper that there is much to learn from previous public health advocacy and action against health-harming industries, such as tobacco, through a ‘Commercial Determinants of Health’ (CDoH) lens. The MPOWER framework is an example of work in tobacco control field which has been a valuable and cross-cutting framework used to guide and support tobacco policy implementation and prioritisation since its development in 2008, contributing to progress in reducing smoking rates and associated morbidity and mortality.

## Background

In 2022, the Lancet Countdown warned that ‘people’s health is at the mercy of fossil fuels’,^1^ yet the health community movement against fossil fuel expansion has never been stronger. That year, 192 health organisations globally, including the World Health Organization (WHO), signed the call for a fossil fuel non-proliferation treaty.^2^ A 2023 open letter from organisations representing 46.3 million health workers helped secure global commitment to transition away from fossil fuels, highlighting the health imperative for their phase-out.^3^

In April 2024, the Faculty of Public Health (FPH) published a Position Statement on Fossil Fuels and Public Health.^4^ It problematises the fossil fuel-dependent energy system as the primary driver of the climate crisis and wider health harms, from risks to frontline community and workers, to air pollution and energy poverty. The statement argues for applying a commercial determinants of health (CDoH) perspective to fossil fuels, understood in this context as the ‘strategies and approaches used by the private sector to promote products and choices that are detrimental to health’.^5^

## The Fossil Fuel Industry as a CDoH

The fossil fuel industry wields power through its influence on global economic, political, and social systems and has historically employed strategies that obstruct environmental and health protection efforts. These include spreading doubt on climate science and lobbying against regulations,^6^ approaches similar to those used by the tobacco and other health-harming industries to delay policy action.^7^ Adopting a CDoH lens helps expose fossil fuel industry tactics and provides a framework for mitigating its influence.^8^ We have therefore developed an adapted version of the WHO’s MPOWER framework for tobacco control,^9^ called MPOWER+, for comprehensive public health action against fossil fuel-related harms.

**Figure:**
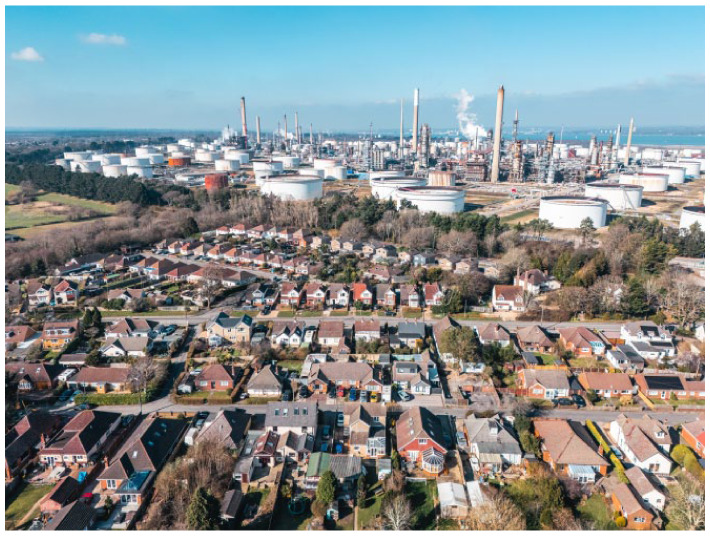


## Mpower for Tobacco Control

**Figure 1.**
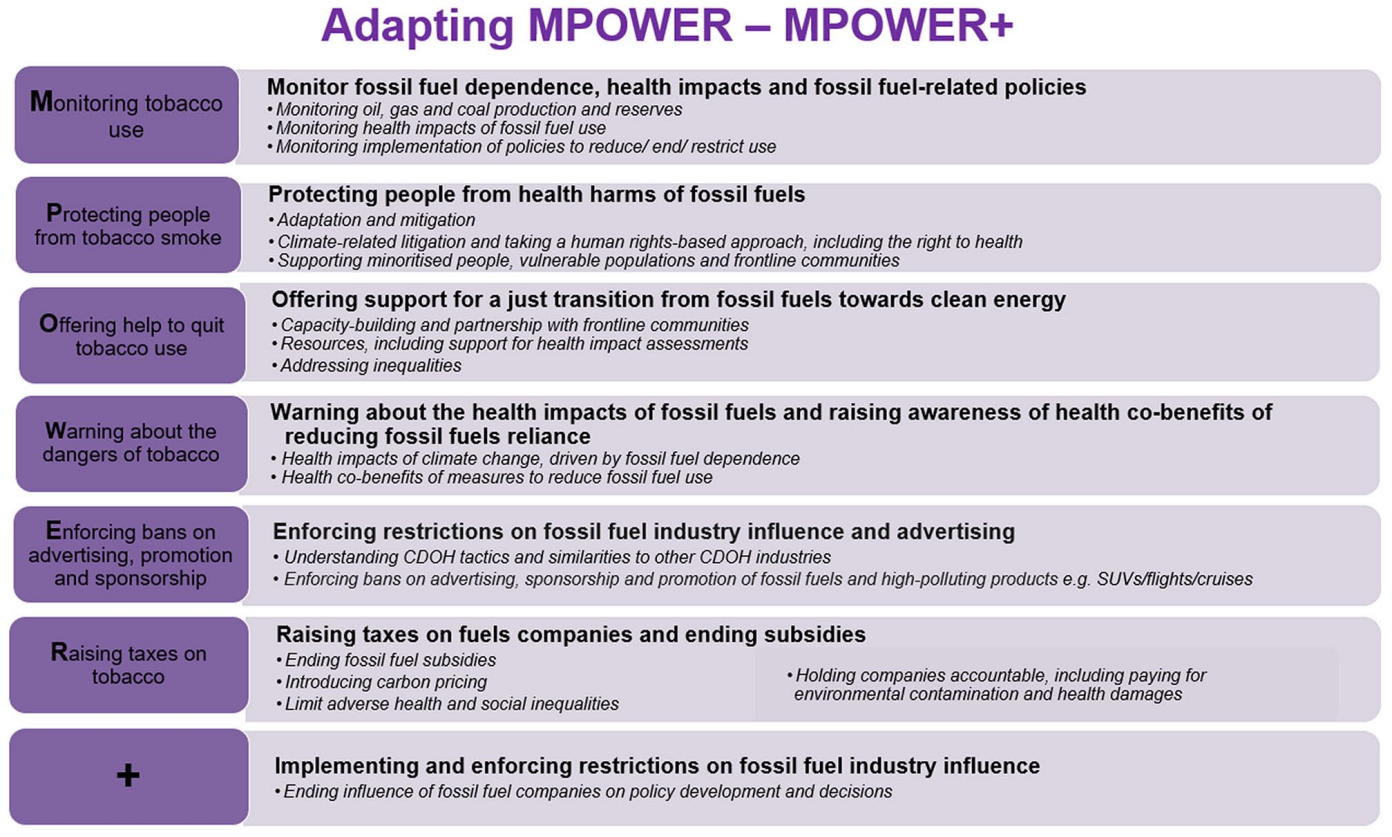
The MPOWER+ framework for fossil fuels.

MPOWER is a framework, developed by the WHO in 2008, to support countries in implementing the Framework Convention on Tobacco Control (FCTC). It provides a coordinated, multi-agency approach by breaking tobacco control into evidence-based steps and policies.^10^ Its components for tobacco control are detailed on the left side of Figure 1, and tobacco control is most effective when all components are enacted. This comprehensive approach has been instrumental in reducing smoking rates globally, helping governments and organisations implement effective and cost-effective policy interventions and monitor their impact.^11,12^

The fossil fuel industry employs similar tactics to the tobacco industry to delay action. It has funded research that downplays climate risks and educational initiatives that shape public discourse,^1^ and has infiltrated policymaking through direct lobbying, sponsorships and revolving-door relationships between government and industry.^8^ Through ‘greenwashing’, corporations may present themselves as environmentally conscious while continuing extractive practices.^13^ In addition, many fossil fuel companies are state-owned, and/or produce some renewable energy, and, unlike tobacco, access to energy is now a fundamental necessity, powering homes, transport, healthcare, and industries, all of which can complicate efforts to challenge fossil fuel companies’ social licence. Public health strategies must not only expose fossil fuel harms but also advocate for a just energy transition – ensuring accessible, sustainable, and healthier energy alternatives.

## Developing MPOWER+ for Fossil Fuels

The adaptation of MPOWER for fossil fuels began with a workshop held by the FPH, focusing on options for implementing policy recommendations from FPH’s Fossil Fuels Position Statement. The aim was to develop an effective CDoH-informed approach to advocacy on fossil fuel-related harms and support a coordinated public health approach to fossil fuels and a just energy transition.

It was agreed that adapting MPOWER, as an overarching, well-recognised, framework that breaks down the issue through tangible actions would be useful for public health professionals across multiple contexts. The MPOWER+ for Fossil Fuels framework was then developed using an expert consensus approach, by

1. mapping options for adapting the framework for fossil fuel advocacy;
2. gathering evidence of the public health case for action and good practice examples, by domain;
3. an online workshop with wider FPH members for further refinement and examples; and
4. incorporating feedback and finalising the framework with additional expert input.

A critical addition to the framework is the ‘+’, which advocates explicitly excluding fossil fuel industry representatives from climate negotiations and health policymaking (e.g. the UNFCCC Conference of Parties), aligned with Article 5.3 of the WHO FCTC.

In developing the MPOWER+ framework, we also reviewed documents including the Lancet CDoH Future Directions Panel^10^ and the recommendations from the International Health and Climate Community for COP29.^14^ The supplementary material details key links identified.

## Limitations and Next Steps

The MPOWER framework for tobacco is a top-down policy framework, not inherently calling for engagement with frontline communities who have a critical role to play in eliminating fossil fuel expansion and working towards a just energy transition. To ensure MPOWER+ responds to the needs of affected populations, bottom-up approaches that prioritise collaboration with most affected communities should be integrated.

In developing MPOWER+, we did not begin with the question of what a comprehensive, evidence-based approach to ending fossil fuel dependency and achieving climate health and equity would involve, meaning that other critical policy issues may be missing. Another complexity to implementing it – especially the ‘+’ – is that, unlike tobacco companies, many national governments are among the world’s largest fossil fuel producers, creating vested interests within policy fora.

MPOWER+ has so far been developed primarily through UK-based public health stakeholders’ input. To strengthen its applicability, further work to extend the framework, including international engagement is necessary. Future steps may include webinars with global health networks to gather feedback and stakeholder collaboration to adapt MPOWER+ for different national contexts.

## Conclusions

The health effects of climate change, particularly those linked to fossil fuel dependency, require a multilevel policy response.^15^ The MPOWER+ Framework translates complex issues into actionable CDoH-informed recommendations. Drawing on lessons from tobacco control, MPOWER+ can serve as a strategic advocacy tool, and a benchmark for tracking progress.

By systematically addressing industry influence and policy gaps, the framework supports public health advocacy for strong, evidence-based policies that end fossil fuel harms and support a just energy transition.

## Supplemental Material

sj-pptx-1-rsh-10.1177_17579139251342161 – Supplemental material for Adapting the MPOWER policy framework for fossil fuels and public health: reflections on content and process Supplemental material, sj-pptx-1-rsh-10.1177_17579139251342161 for Adapting the MPOWER policy framework for fossil fuels and public health: reflections on content and process by T Keller, I Braithwaite, A Brook, J Beagley, S Narayan and TA Deivanayagam in Perspectives in Public Health
