# Supplementary material for: Adapting the MPOWER policy framework for fossil fuels and public health: reflections on content and process
Source: Perspect Public Health. 2025 Jun 28;145(6):302–5. doi: 10.1177/17579139251342161 (PMC12662816; doi:10.1177/17579139251342161)
Supplement: sj-pptx-1-rsh-10.1177_17579139251342161 – Supplemental material for Adapting the MPOWER policy framework for fossil fuels and public health: reflections on content and process [file sj-pptx-1-rsh-10.1177_17579139251342161.pptx]

## Slide 1
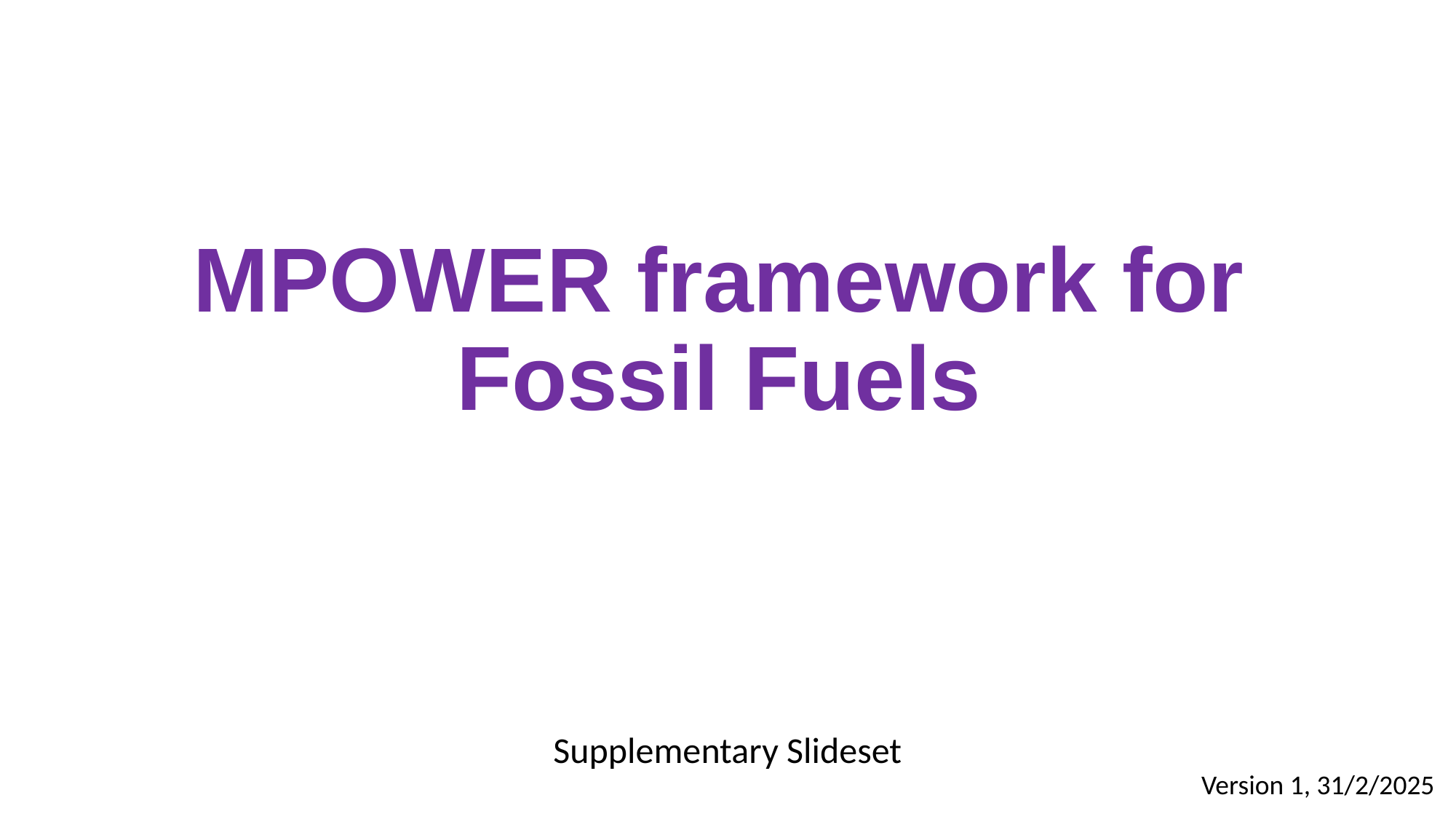

# MPOWER framework for Fossil Fuels
Supplementary Slideset
Version 1, 31/2/2025

## Slide 2
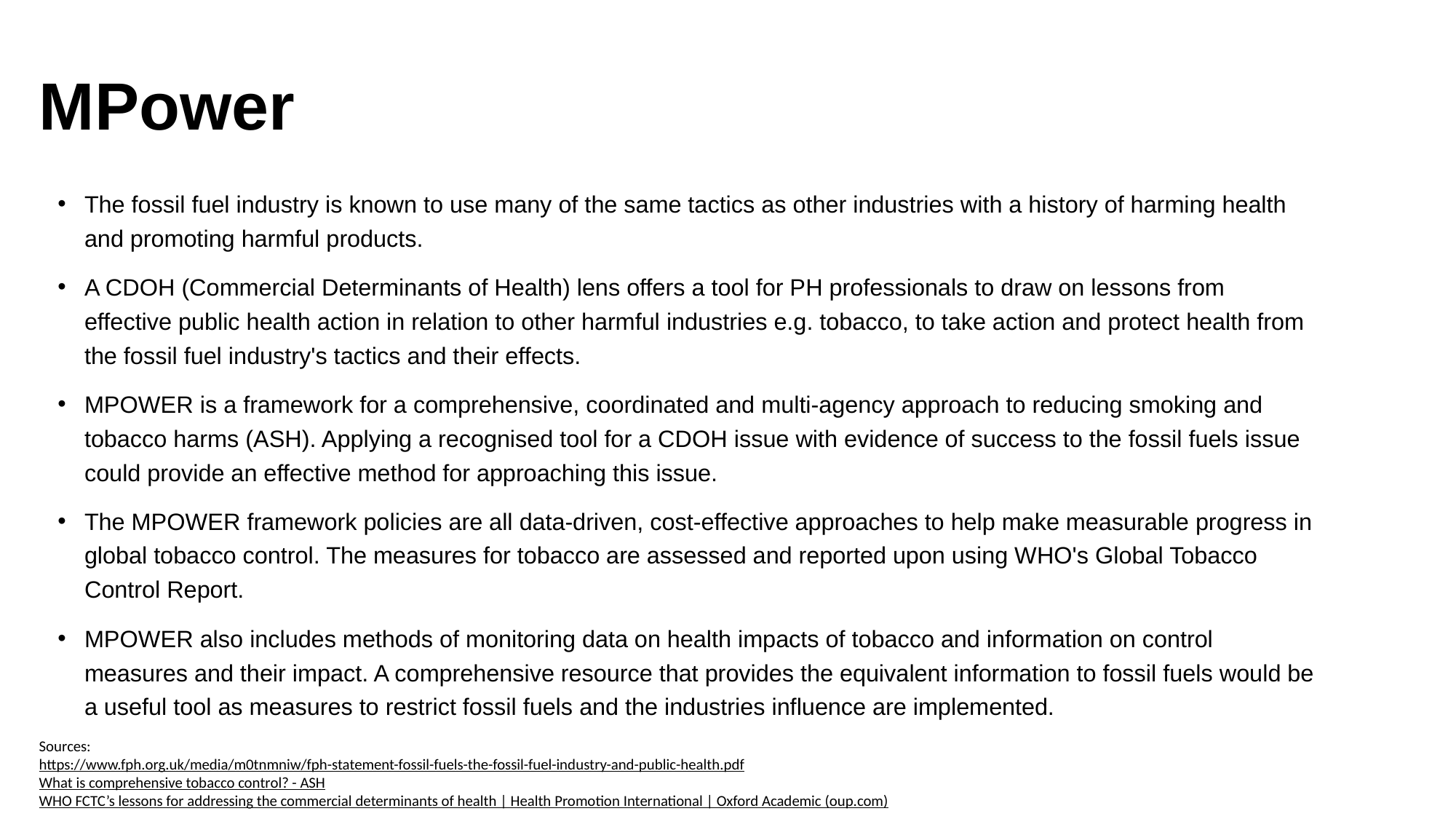

# MPower
The fossil fuel industry is known to use many of the same tactics as other industries with a history of harming health and promoting harmful products.
A CDOH (Commercial Determinants of Health) lens offers a tool for PH professionals to draw on lessons from effective public health action in relation to other harmful industries e.g. tobacco, to take action and protect health from the fossil fuel industry's tactics and their effects.
MPOWER is a framework for a comprehensive, coordinated and multi-agency approach to reducing smoking and tobacco harms (ASH). Applying a recognised tool for a CDOH issue with evidence of success to the fossil fuels issue could provide an effective method for approaching this issue.
The MPOWER framework policies are all data-driven, cost-effective approaches to help make measurable progress in global tobacco control. The measures for tobacco are assessed and reported upon using WHO's Global Tobacco Control Report.
MPOWER also includes methods of monitoring data on health impacts of tobacco and information on control measures and their impact. A comprehensive resource that provides the equivalent information to fossil fuels would be a useful tool as measures to restrict fossil fuels and the industries influence are implemented.
Sources:
https://www.fph.org.uk/media/m0tnmniw/fph-statement-fossil-fuels-the-fossil-fuel-industry-and-public-health.pdf
What is comprehensive tobacco control? - ASH
WHO FCTC’s lessons for addressing the commercial determinants of health | Health Promotion International | Oxford Academic (oup.com)

## Slide 3
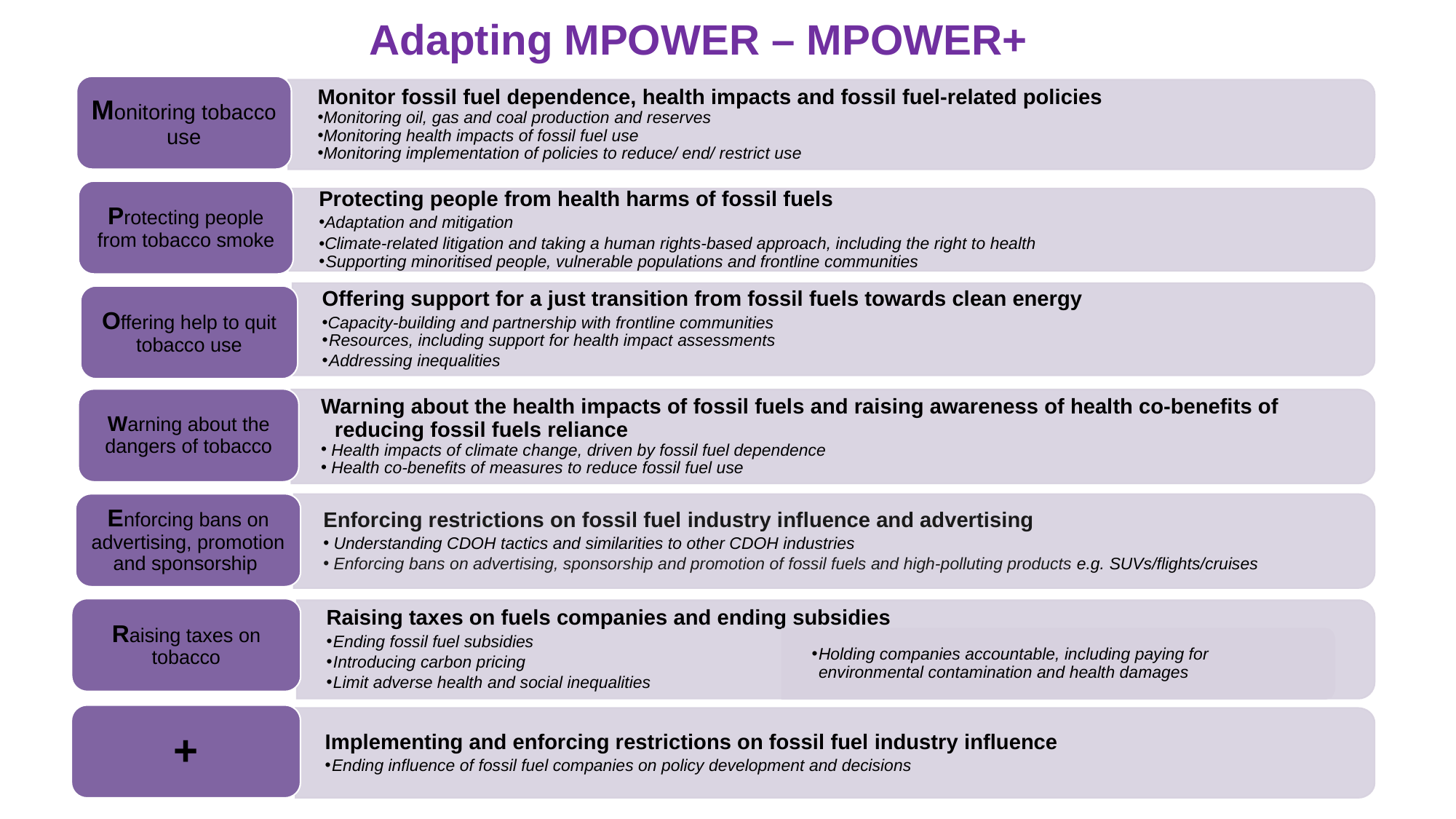

# Adapting MPOWER – MPOWER+
Monitoring tobacco use
Monitor fossil fuel dependence, health impacts and fossil fuel-related policies
Monitoring oil, gas and coal production and reserves
Monitoring health impacts of fossil fuel use
Monitoring implementation of policies to reduce/ end/ restrict use
Protecting people from tobacco smoke
Protecting people from health harms of fossil fuels
Adaptation and mitigation
Climate-related litigation and taking a human rights-based approach, including the right to health
Supporting minoritised people, vulnerable populations and frontline communities
Offering support for a just transition from fossil fuels towards clean energy
Capacity-building and partnership with frontline communities
Resources, including support for health impact assessments
Addressing inequalities
Offering help to quit tobacco use
Warning about the dangers of tobacco
Warning about the health impacts of fossil fuels and raising awareness of health co-benefits of reducing fossil fuels reliance
Health impacts of climate change, driven by fossil fuel dependence
Health co-benefits of measures to reduce fossil fuel use
Enforcing bans on advertising, promotion and sponsorship
Enforcing restrictions on fossil fuel industry influence and advertising
Understanding CDOH tactics and similarities to other CDOH industries
Enforcing bans on advertising, sponsorship and promotion of fossil fuels and high-polluting products e.g. SUVs/flights/cruises
Raising taxes on tobacco
Raising taxes on fuels companies and ending subsidies
Ending fossil fuel subsidies
Introducing carbon pricing
Limit adverse health and social inequalities
Holding companies accountable, including paying for environmental contamination and health damages
+
Implementing and enforcing restrictions on fossil fuel industry influence
Ending influence of fossil fuel companies on policy development and decisions

## Slide 4
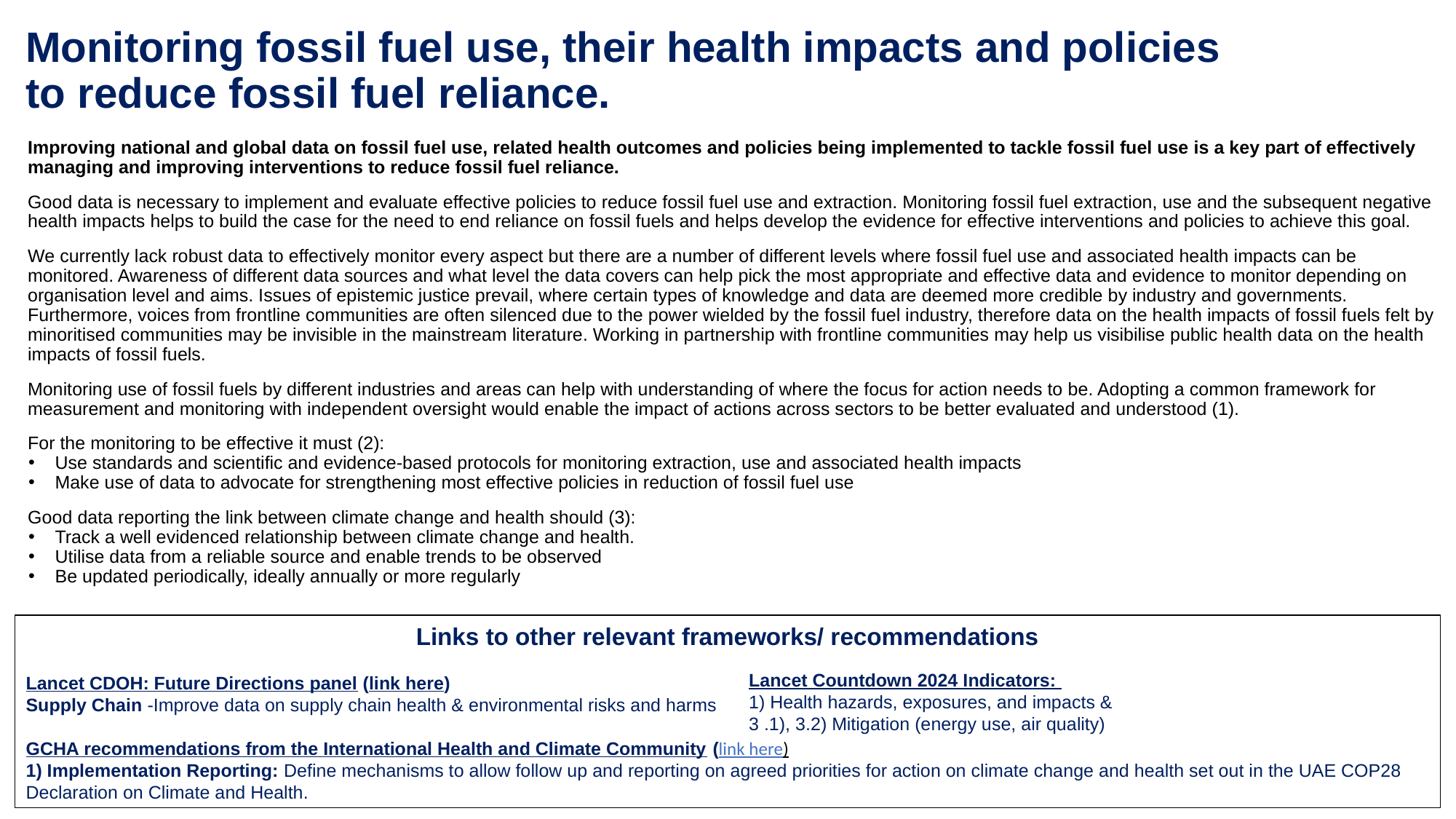

Monitoring fossil fuel use, their health impacts and policies to reduce fossil fuel reliance.
Improving national and global data on fossil fuel use, related health outcomes and policies being implemented to tackle fossil fuel use is a key part of effectively managing and improving interventions to reduce fossil fuel reliance.
Good data is necessary to implement and evaluate effective policies to reduce fossil fuel use and extraction. Monitoring fossil fuel extraction, use and the subsequent negative health impacts helps to build the case for the need to end reliance on fossil fuels and helps develop the evidence for effective interventions and policies to achieve this goal.
We currently lack robust data to effectively monitor every aspect but there are a number of different levels where fossil fuel use and associated health impacts can be monitored. Awareness of different data sources and what level the data covers can help pick the most appropriate and effective data and evidence to monitor depending on organisation level and aims. Issues of epistemic justice prevail, where certain types of knowledge and data are deemed more credible by industry and governments. Furthermore, voices from frontline communities are often silenced due to the power wielded by the fossil fuel industry, therefore data on the health impacts of fossil fuels felt by minoritised communities may be invisible in the mainstream literature. Working in partnership with frontline communities may help us visibilise public health data on the health impacts of fossil fuels.
Monitoring use of fossil fuels by different industries and areas can help with understanding of where the focus for action needs to be. Adopting a common framework for measurement and monitoring with independent oversight would enable the impact of actions across sectors to be better evaluated and understood (1).
For the monitoring to be effective it must (2):
Use standards and scientific and evidence-based protocols for monitoring extraction, use and associated health impacts
Make use of data to advocate for strengthening most effective policies in reduction of fossil fuel use
Good data reporting the link between climate change and health should (3):
Track a well evidenced relationship between climate change and health.
Utilise data from a reliable source and enable trends to be observed
Be updated periodically, ideally annually or more regularly
Links to other relevant frameworks/ recommendations
Lancet CDOH: Future Directions panel (link here)
Supply Chain -Improve data on supply chain health & environmental risks and harms
GCHA recommendations from the International Health and Climate Community (link here)
1) Implementation Reporting: Define mechanisms to allow follow up and reporting on agreed priorities for action on climate change and health set out in the UAE COP28 Declaration on Climate and Health.
Lancet Countdown 2024 Indicators:
1) Health hazards, exposures, and impacts &
3 .1), 3.2) Mitigation (energy use, air quality)

## Slide 5
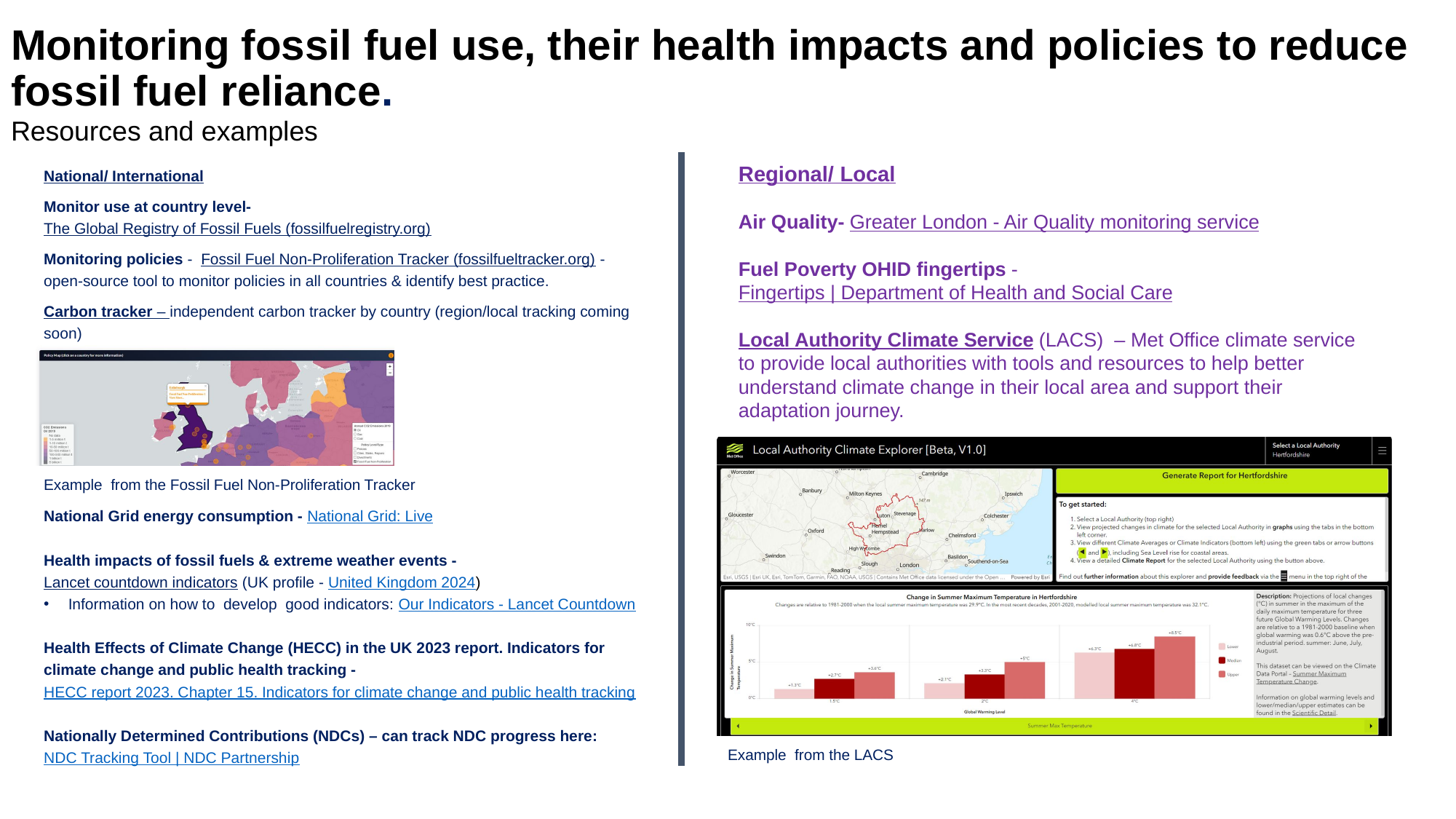

# Monitoring fossil fuel use, their health impacts and policies to reduce fossil fuel reliance.
Resources and examples
National/ International
Monitor use at country level- The Global Registry of Fossil Fuels (fossilfuelregistry.org)
Monitoring policies - Fossil Fuel Non-Proliferation Tracker (fossilfueltracker.org) - open-source tool to monitor policies in all countries & identify best practice.
Carbon tracker – independent carbon tracker by country (region/local tracking coming soon)
National Grid energy consumption - National Grid: Live
Health impacts of fossil fuels & extreme weather events - Lancet countdown indicators (UK profile - United Kingdom 2024)
Information on how to develop good indicators: Our Indicators - Lancet Countdown
Health Effects of Climate Change (HECC) in the UK 2023 report. Indicators for climate change and public health tracking - HECC report 2023. Chapter 15. Indicators for climate change and public health tracking
Nationally Determined Contributions (NDCs) – can track NDC progress here: NDC Tracking Tool | NDC Partnership
Regional/ Local
Air Quality- Greater London - Air Quality monitoring service
Fuel Poverty OHID fingertips - Fingertips | Department of Health and Social Care
Local Authority Climate Service (LACS) – Met Office climate service to provide local authorities with tools and resources to help better understand climate change in their local area and support their adaptation journey.
Example from the Fossil Fuel Non-Proliferation Tracker
Example from the LACS

## Slide 6
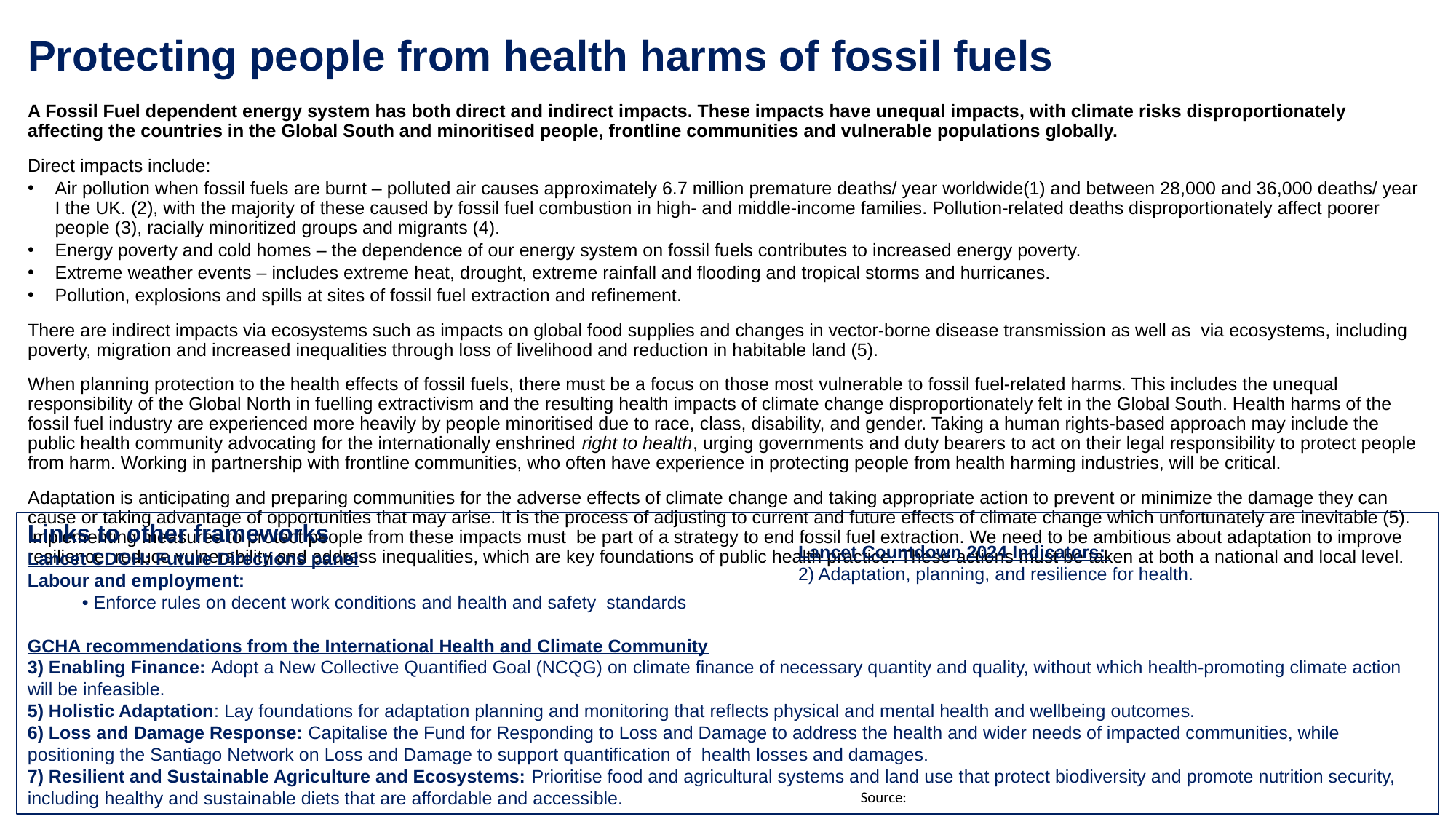

# Protecting people from health harms of fossil fuels
A Fossil Fuel dependent energy system has both direct and indirect impacts. These impacts have unequal impacts, with climate risks disproportionately affecting the countries in the Global South and minoritised people, frontline communities and vulnerable populations globally.
Direct impacts include:
Air pollution when fossil fuels are burnt – polluted air causes approximately 6.7 million premature deaths/ year worldwide(1) and between 28,000 and 36,000 deaths/ year I the UK. (2), with the majority of these caused by fossil fuel combustion in high- and middle-income families. Pollution-related deaths disproportionately affect poorer people (3), racially minoritized groups and migrants (4).
Energy poverty and cold homes – the dependence of our energy system on fossil fuels contributes to increased energy poverty.
Extreme weather events – includes extreme heat, drought, extreme rainfall and flooding and tropical storms and hurricanes.
Pollution, explosions and spills at sites of fossil fuel extraction and refinement.
There are indirect impacts via ecosystems such as impacts on global food supplies and changes in vector-borne disease transmission as well as via ecosystems, including poverty, migration and increased inequalities through loss of livelihood and reduction in habitable land (5).
When planning protection to the health effects of fossil fuels, there must be a focus on those most vulnerable to fossil fuel-related harms. This includes the unequal responsibility of the Global North in fuelling extractivism and the resulting health impacts of climate change disproportionately felt in the Global South. Health harms of the fossil fuel industry are experienced more heavily by people minoritised due to race, class, disability, and gender. Taking a human rights-based approach may include the public health community advocating for the internationally enshrined right to health, urging governments and duty bearers to act on their legal responsibility to protect people from harm. Working in partnership with frontline communities, who often have experience in protecting people from health harming industries, will be critical.
Adaptation is anticipating and preparing communities for the adverse effects of climate change and taking appropriate action to prevent or minimize the damage they can cause or taking advantage of opportunities that may arise. It is the process of adjusting to current and future effects of climate change which unfortunately are inevitable (5). Implementing measures to protect people from these impacts must be part of a strategy to end fossil fuel extraction. We need to be ambitious about adaptation to improve resilience, reduce vulnerability and address inequalities, which are key foundations of public health practice. These actions must be taken at both a national and local level.
Links to other frameworks
Lancet CDOH: Future Directions panel
Labour and employment:
• Enforce rules on decent work conditions and health and safety standards
GCHA recommendations from the International Health and Climate Community
3) Enabling Finance: Adopt a New Collective Quantified Goal (NCQG) on climate finance of necessary quantity and quality, without which health-promoting climate action will be infeasible.
5) Holistic Adaptation: Lay foundations for adaptation planning and monitoring that reflects physical and mental health and wellbeing outcomes.
6) Loss and Damage Response: Capitalise the Fund for Responding to Loss and Damage to address the health and wider needs of impacted communities, while positioning the Santiago Network on Loss and Damage to support quantification of  health losses and damages.
7) Resilient and Sustainable Agriculture and Ecosystems: Prioritise food and agricultural systems and land use that protect biodiversity and promote nutrition security, including healthy and sustainable diets that are affordable and accessible.
Lancet Countdown 2024 Indicators:
2) Adaptation, planning, and resilience for health.
Source:

## Slide 7
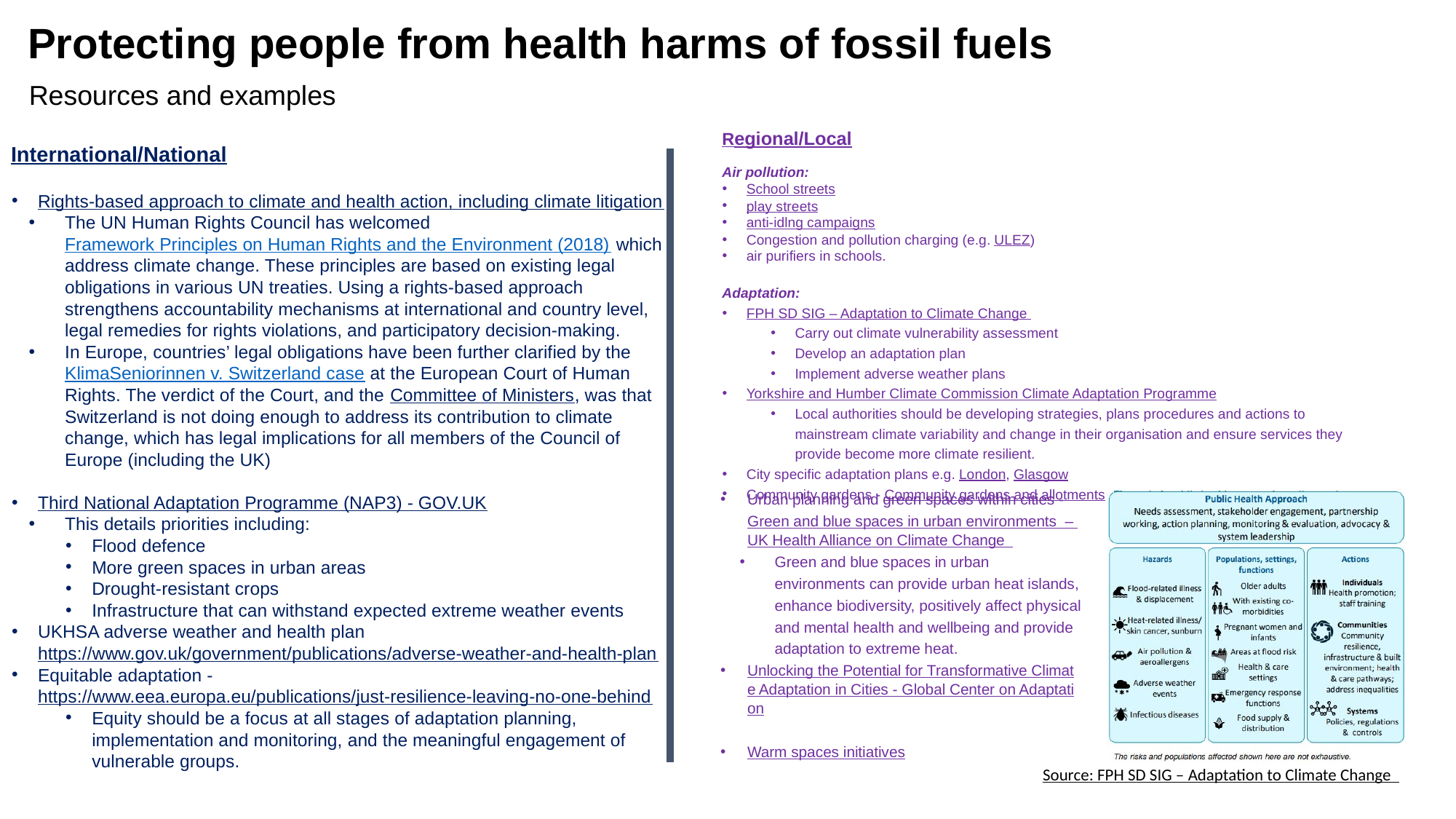

# Protecting people from health harms of fossil fuels
Resources and examples
Regional/Local
Air pollution:
School streets
play streets
anti-idlng campaigns
Congestion and pollution charging (e.g. ULEZ)
air purifiers in schools.
Adaptation:
FPH SD SIG – Adaptation to Climate Change
Carry out climate vulnerability assessment
Develop an adaptation plan
Implement adverse weather plans
Yorkshire and Humber Climate Commission Climate Adaptation Programme
Local authorities should be developing strategies, plans procedures and actions to mainstream climate variability and change in their organisation and ensure services they provide become more climate resilient.
City specific adaptation plans e.g. London, Glasgow
Community gardens - Community gardens and allotments
Urban planning and green spaces within cities - Green and blue spaces in urban environments – UK Health Alliance on Climate Change
Green and blue spaces in urban environments can provide urban heat islands, enhance biodiversity, positively affect physical and mental health and wellbeing and provide adaptation to extreme heat.
Unlocking the Potential for Transformative Climate Adaptation in Cities - Global Center on Adaptation
Warm spaces initiatives
International/National
Rights-based approach to climate and health action, including climate litigation
The UN Human Rights Council has welcomed Framework Principles on Human Rights and the Environment (2018) which address climate change. These principles are based on existing legal obligations in various UN treaties. Using a rights-based approach strengthens accountability mechanisms at international and country level, legal remedies for rights violations, and participatory decision-making.
In Europe, countries’ legal obligations have been further clarified by the KlimaSeniorinnen v. Switzerland case at the European Court of Human Rights. The verdict of the Court, and the Committee of Ministers, was that Switzerland is not doing enough to address its contribution to climate change, which has legal implications for all members of the Council of Europe (including the UK)
Third National Adaptation Programme (NAP3) - GOV.UK
This details priorities including:
Flood defence
More green spaces in urban areas
Drought-resistant crops
Infrastructure that can withstand expected extreme weather events
UKHSA adverse weather and health plan https://www.gov.uk/government/publications/adverse-weather-and-health-plan
Equitable adaptation - https://www.eea.europa.eu/publications/just-resilience-leaving-no-one-behind
Equity should be a focus at all stages of adaptation planning, implementation and monitoring, and the meaningful engagement of vulnerable groups.
Source: FPH SD SIG – Adaptation to Climate Change

## Slide 8
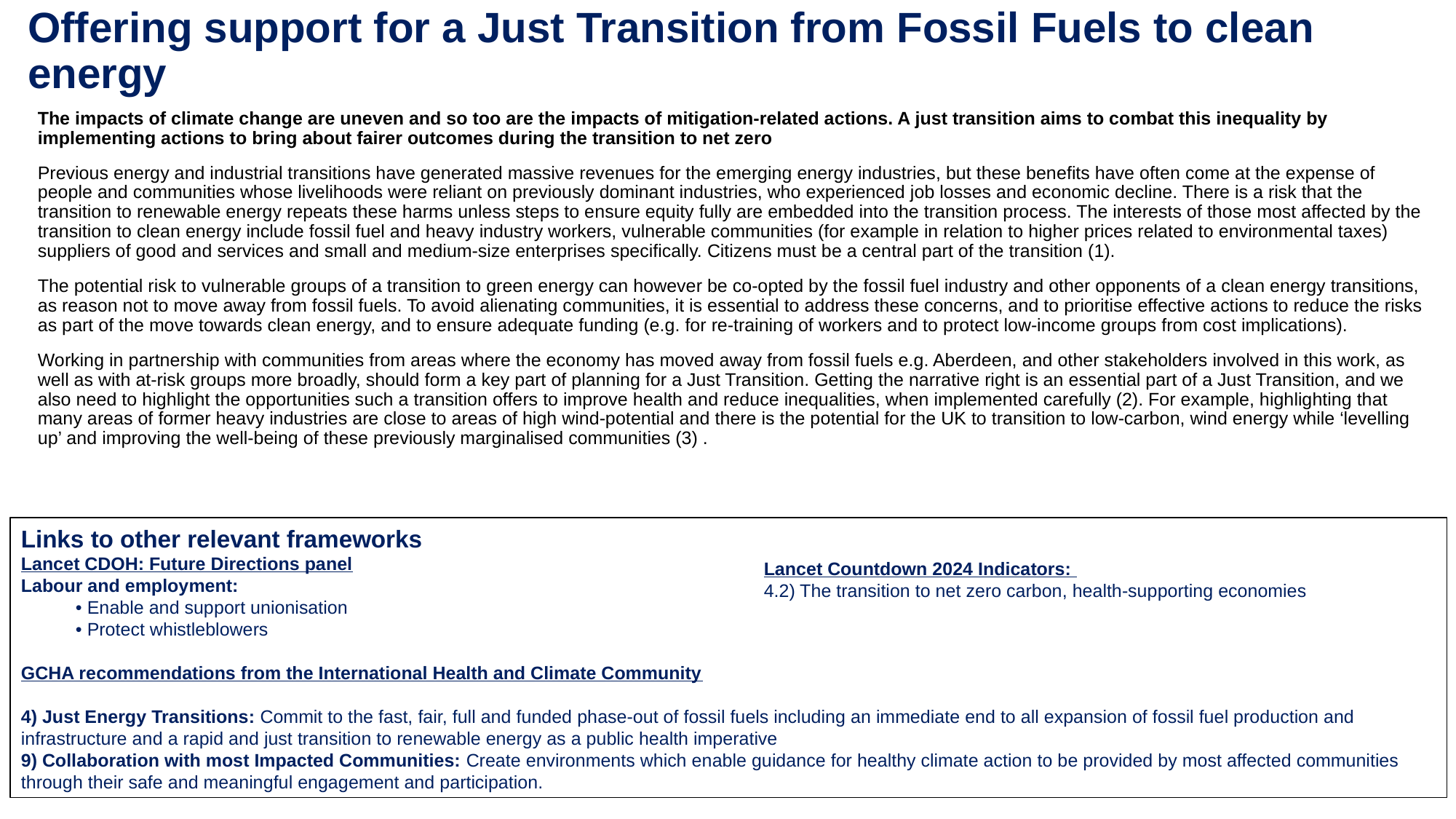

# Offering support for a Just Transition from Fossil Fuels to clean energy
The impacts of climate change are uneven and so too are the impacts of mitigation-related actions. A just transition aims to combat this inequality by implementing actions to bring about fairer outcomes during the transition to net zero
Previous energy and industrial transitions have generated massive revenues for the emerging energy industries, but these benefits have often come at the expense of people and communities whose livelihoods were reliant on previously dominant industries, who experienced job losses and economic decline. There is a risk that the transition to renewable energy repeats these harms unless steps to ensure equity fully are embedded into the transition process. The interests of those most affected by the transition to clean energy include fossil fuel and heavy industry workers, vulnerable communities (for example in relation to higher prices related to environmental taxes) suppliers of good and services and small and medium-size enterprises specifically. Citizens must be a central part of the transition (1).
The potential risk to vulnerable groups of a transition to green energy can however be co-opted by the fossil fuel industry and other opponents of a clean energy transitions, as reason not to move away from fossil fuels. To avoid alienating communities, it is essential to address these concerns, and to prioritise effective actions to reduce the risks as part of the move towards clean energy, and to ensure adequate funding (e.g. for re-training of workers and to protect low-income groups from cost implications).
Working in partnership with communities from areas where the economy has moved away from fossil fuels e.g. Aberdeen, and other stakeholders involved in this work, as well as with at-risk groups more broadly, should form a key part of planning for a Just Transition. Getting the narrative right is an essential part of a Just Transition, and we also need to highlight the opportunities such a transition offers to improve health and reduce inequalities, when implemented carefully (2). For example, highlighting that many areas of former heavy industries are close to areas of high wind-potential and there is the potential for the UK to transition to low-carbon, wind energy while ‘levelling up’ and improving the well-being of these previously marginalised communities (3) .
Links to other relevant frameworks
Lancet CDOH: Future Directions panel
Labour and employment:
• Enable and support unionisation
• Protect whistleblowers
GCHA recommendations from the International Health and Climate Community
4) Just Energy Transitions: Commit to the fast, fair, full and funded phase-out of fossil fuels including an immediate end to all expansion of fossil fuel production and infrastructure and a rapid and just transition to renewable energy as a public health imperative
9) Collaboration with most Impacted Communities: Create environments which enable guidance for healthy climate action to be provided by most affected communities through their safe and meaningful engagement and participation.
Lancet Countdown 2024 Indicators:
4.2) The transition to net zero carbon, health-supporting economies

## Slide 9
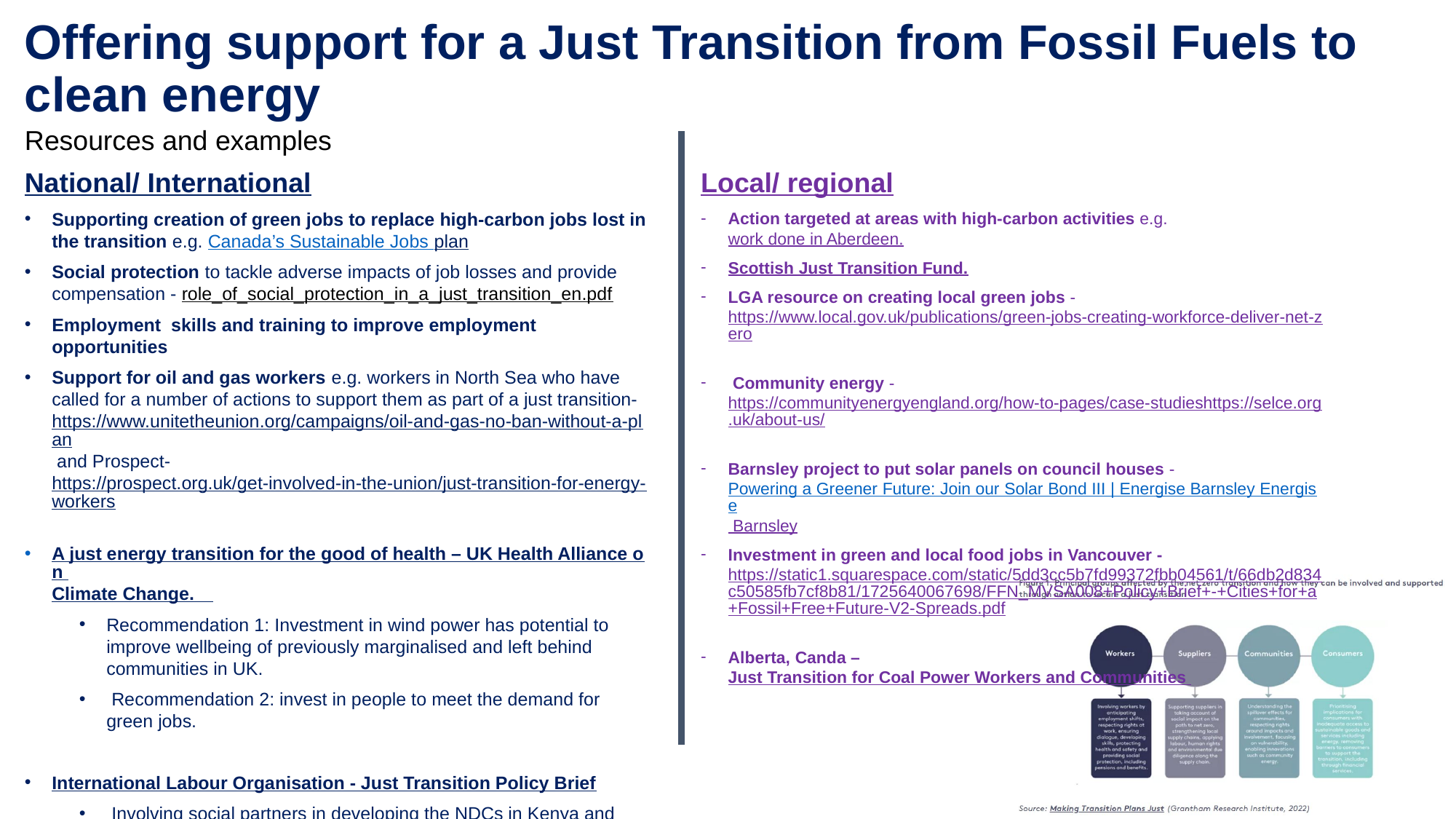

# Offering support for a Just Transition from Fossil Fuels to clean energy
Resources and examples
National/ International
Supporting creation of green jobs to replace high-carbon jobs lost in the transition e.g. Canada’s Sustainable Jobs plan
Social protection to tackle adverse impacts of job losses and provide compensation - role_of_social_protection_in_a_just_transition_en.pdf
Employment skills and training to improve employment opportunities
Support for oil and gas workers e.g. workers in North Sea who have called for a number of actions to support them as part of a just transition- https://www.unitetheunion.org/campaigns/oil-and-gas-no-ban-without-a-plan and Prospect- https://prospect.org.uk/get-involved-in-the-union/just-transition-for-energy-workers
A just energy transition for the good of health – UK Health Alliance on Climate Change.
Recommendation 1: Investment in wind power has potential to improve wellbeing of previously marginalised and left behind communities in UK.
 Recommendation 2: invest in people to meet the demand for green jobs.
International Labour Organisation - Just Transition Policy Brief
 Involving social partners in developing the NDCs in Kenya and Costa Rica.
Local/ regional
Action targeted at areas with high-carbon activities e.g. work done in Aberdeen.
Scottish Just Transition Fund.
LGA resource on creating local green jobs - https://www.local.gov.uk/publications/green-jobs-creating-workforce-deliver-net-zero
 Community energy - https://communityenergyengland.org/how-to-pages/case-studieshttps://selce.org.uk/about-us/
Barnsley project to put solar panels on council houses -Powering a Greener Future: Join our Solar Bond III | Energise Barnsley Energise Barnsley
Investment in green and local food jobs in Vancouver - https://static1.squarespace.com/static/5dd3cc5b7fd99372fbb04561/t/66db2d834c50585fb7cf8b81/1725640067698/FFN_MVSA008+Policy+Brief+-+Cities+for+a+Fossil+Free+Future-V2-Spreads.pdf
Alberta, Canda – Just Transition for Coal Power Workers and Communities

## Slide 10
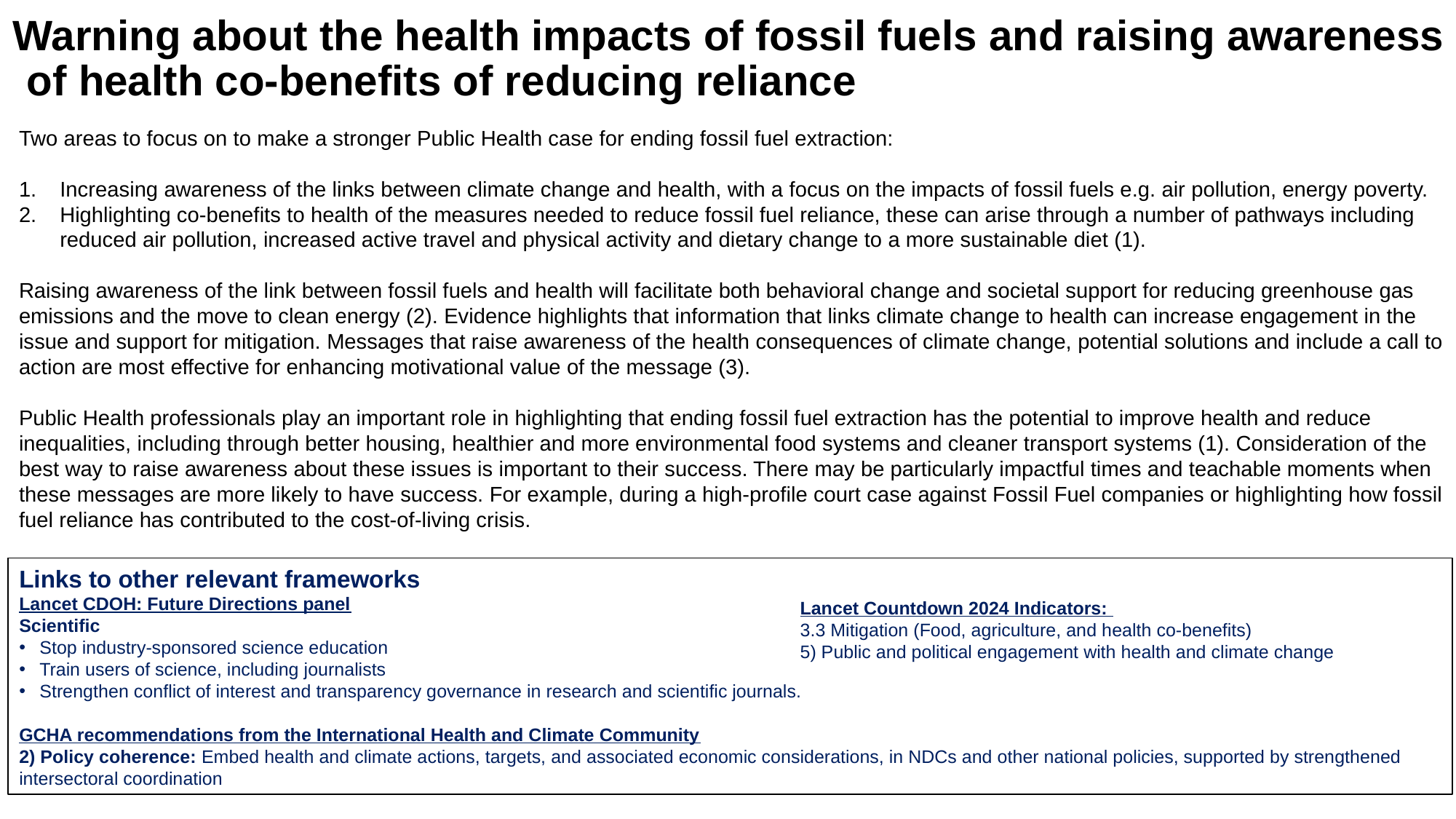

# Warning about the health impacts of fossil fuels and raising awareness of health co-benefits of reducing reliance
Two areas to focus on to make a stronger Public Health case for ending fossil fuel extraction:
Increasing awareness of the links between climate change and health, with a focus on the impacts of fossil fuels e.g. air pollution, energy poverty.
Highlighting co-benefits to health of the measures needed to reduce fossil fuel reliance, these can arise through a number of pathways including reduced air pollution, increased active travel and physical activity and dietary change to a more sustainable diet (1).
Raising awareness of the link between fossil fuels and health will facilitate both behavioral change and societal support for reducing greenhouse gas emissions and the move to clean energy (2). Evidence highlights that information that links climate change to health can increase engagement in the issue and support for mitigation. Messages that raise awareness of the health consequences of climate change, potential solutions and include a call to action are most effective for enhancing motivational value of the message (3).
Public Health professionals play an important role in highlighting that ending fossil fuel extraction has the potential to improve health and reduce inequalities, including through better housing, healthier and more environmental food systems and cleaner transport systems (1). Consideration of the best way to raise awareness about these issues is important to their success. There may be particularly impactful times and teachable moments when these messages are more likely to have success. For example, during a high-profile court case against Fossil Fuel companies or highlighting how fossil fuel reliance has contributed to the cost-of-living crisis.
Links to other relevant frameworks
Lancet CDOH: Future Directions panel
Scientific
Stop industry-sponsored science education
Train users of science, including journalists
Strengthen conflict of interest and transparency governance in research and scientific journals.
GCHA recommendations from the International Health and Climate Community
2) Policy coherence: Embed health and climate actions, targets, and associated economic considerations, in NDCs and other national policies, supported by strengthened intersectoral coordination
Lancet Countdown 2024 Indicators:
3.3 Mitigation (Food, agriculture, and health co-benefits)
5) Public and political engagement with health and climate change

## Slide 11
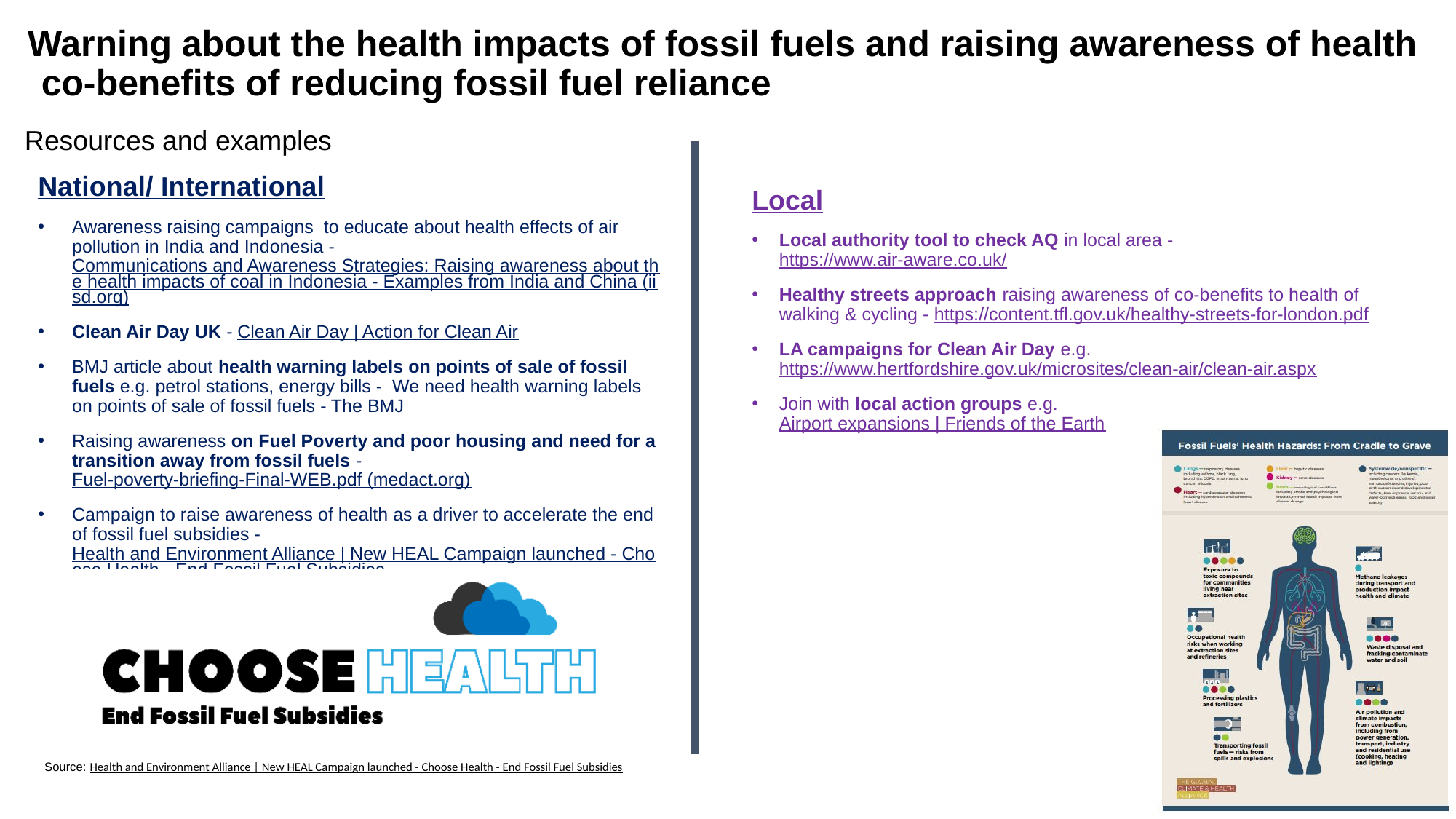

# Warning about the health impacts of fossil fuels and raising awareness of health co-benefits of reducing fossil fuel reliance
Resources and examples
Local
Local authority tool to check AQ in local area - https://www.air-aware.co.uk/
Healthy streets approach raising awareness of co-benefits to health of walking & cycling - https://content.tfl.gov.uk/healthy-streets-for-london.pdf
LA campaigns for Clean Air Day e.g. https://www.hertfordshire.gov.uk/microsites/clean-air/clean-air.aspx
Join with local action groups e.g. Airport expansions | Friends of the Earth
National/ International
Awareness raising campaigns to educate about health effects of air pollution in India and Indonesia - Communications and Awareness Strategies: Raising awareness about the health impacts of coal in Indonesia - Examples from India and China (iisd.org)
Clean Air Day UK - Clean Air Day | Action for Clean Air
BMJ article about health warning labels on points of sale of fossil fuels e.g. petrol stations, energy bills - We need health warning labels on points of sale of fossil fuels - The BMJ
Raising awareness on Fuel Poverty and poor housing and need for a transition away from fossil fuels - Fuel-poverty-briefing-Final-WEB.pdf (medact.org)
Campaign to raise awareness of health as a driver to accelerate the end of fossil fuel subsidies - Health and Environment Alliance | New HEAL Campaign launched - Choose Health - End Fossil Fuel Subsidies
Source: Health and Environment Alliance | New HEAL Campaign launched - Choose Health - End Fossil Fuel Subsidies
Source: Cradle-To-Grave-Fossil-Fuels-Brief.pdf (climateandhealthalliance.org)

## Slide 12
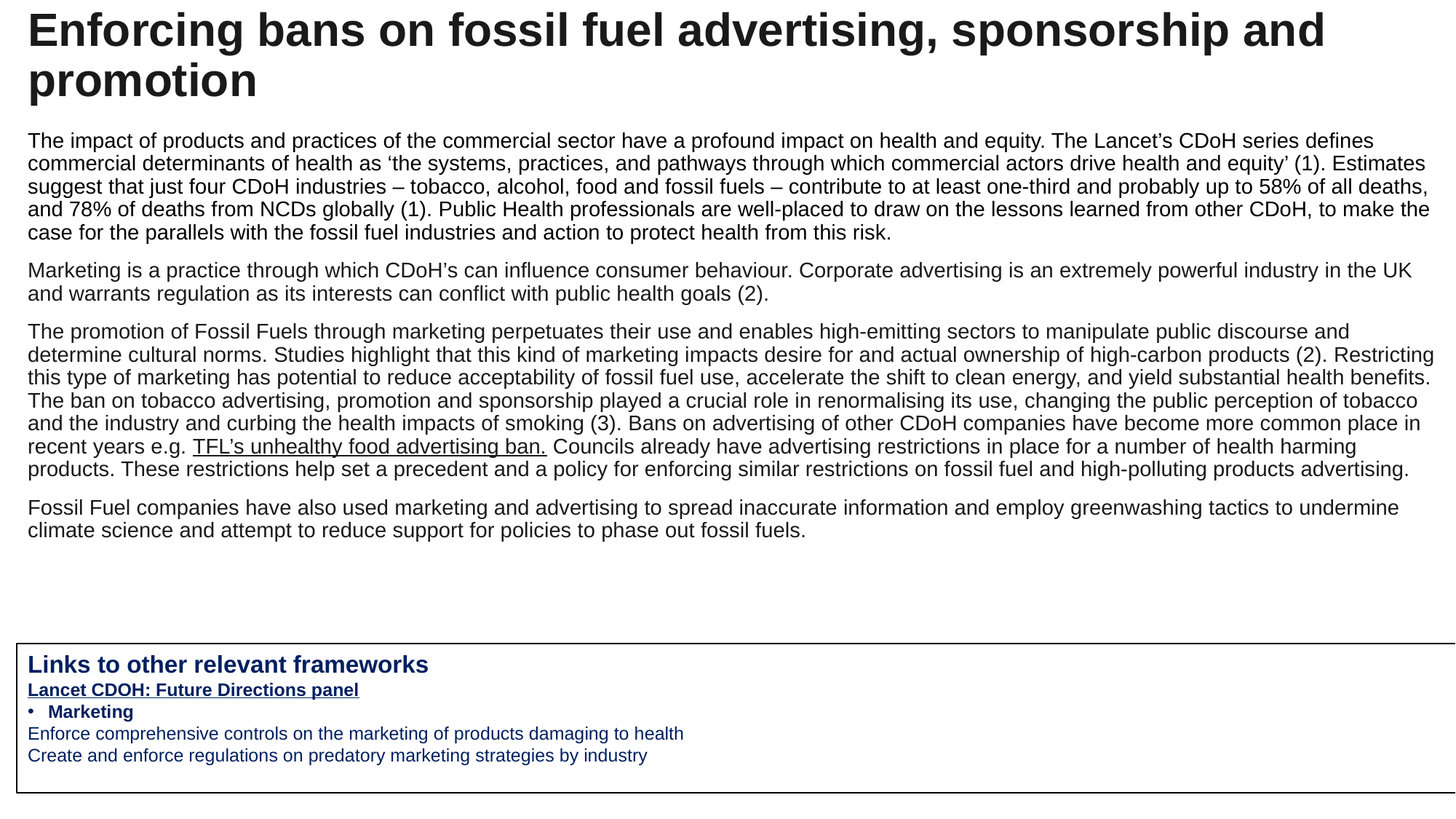

# Enforcing bans on fossil fuel advertising, sponsorship and promotion
The impact of products and practices of the commercial sector have a profound impact on health and equity. The Lancet’s CDoH series defines commercial determinants of health as ‘the systems, practices, and pathways through which commercial actors drive health and equity’ (1). Estimates suggest that just four CDoH industries – tobacco, alcohol, food and fossil fuels – contribute to at least one-third and probably up to 58% of all deaths, and 78% of deaths from NCDs globally (1). Public Health professionals are well-placed to draw on the lessons learned from other CDoH, to make the case for the parallels with the fossil fuel industries and action to protect health from this risk.
Marketing is a practice through which CDoH’s can influence consumer behaviour. Corporate advertising is an extremely powerful industry in the UK and warrants regulation as its interests can conflict with public health goals (2).
The promotion of Fossil Fuels through marketing perpetuates their use and enables high-emitting sectors to manipulate public discourse and determine cultural norms. Studies highlight that this kind of marketing impacts desire for and actual ownership of high-carbon products (2). Restricting this type of marketing has potential to reduce acceptability of fossil fuel use, accelerate the shift to clean energy, and yield substantial health benefits. The ban on tobacco advertising, promotion and sponsorship played a crucial role in renormalising its use, changing the public perception of tobacco and the industry and curbing the health impacts of smoking (3). Bans on advertising of other CDoH companies have become more common place in recent years e.g. TFL’s unhealthy food advertising ban. Councils already have advertising restrictions in place for a number of health harming products. These restrictions help set a precedent and a policy for enforcing similar restrictions on fossil fuel and high-polluting products advertising.
Fossil Fuel companies have also used marketing and advertising to spread inaccurate information and employ greenwashing tactics to undermine climate science and attempt to reduce support for policies to phase out fossil fuels.
Links to other relevant frameworks
Lancet CDOH: Future Directions panel
Marketing
Enforce comprehensive controls on the marketing of products damaging to health
Create and enforce regulations on predatory marketing strategies by industry

## Slide 13
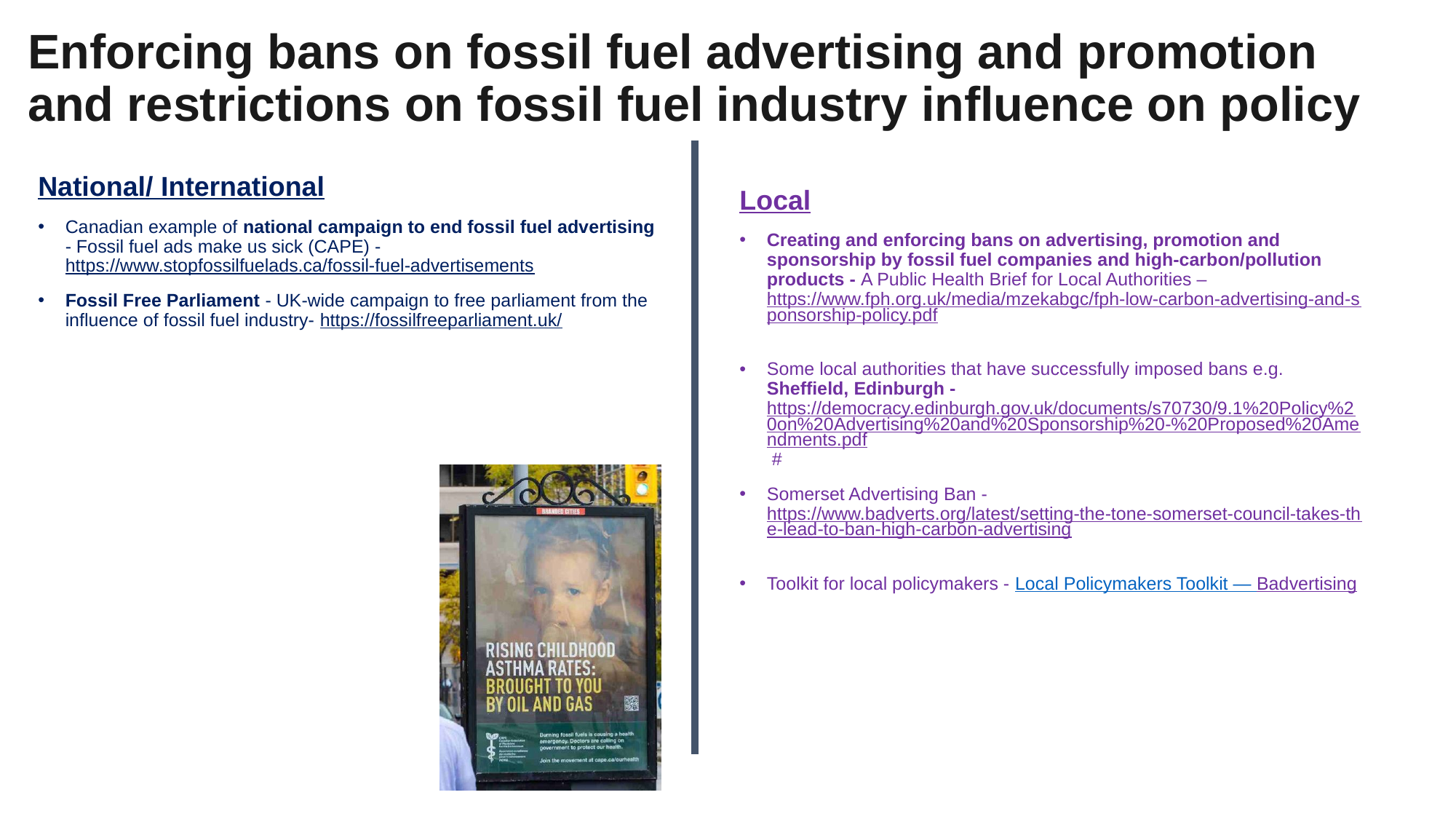

# Enforcing bans on fossil fuel advertising and promotion and restrictions on fossil fuel industry influence on policy
Local
Creating and enforcing bans on advertising, promotion and sponsorship by fossil fuel companies and high-carbon/pollution products - A Public Health Brief for Local Authorities – https://www.fph.org.uk/media/mzekabgc/fph-low-carbon-advertising-and-sponsorship-policy.pdf
Some local authorities that have successfully imposed bans e.g. Sheffield, Edinburgh - https://democracy.edinburgh.gov.uk/documents/s70730/9.1%20Policy%20on%20Advertising%20and%20Sponsorship%20-%20Proposed%20Amendments.pdf #
Somerset Advertising Ban - https://www.badverts.org/latest/setting-the-tone-somerset-council-takes-the-lead-to-ban-high-carbon-advertising
Toolkit for local policymakers - Local Policymakers Toolkit — Badvertising
National/ International
Canadian example of national campaign to end fossil fuel advertising - Fossil fuel ads make us sick (CAPE) -https://www.stopfossilfuelads.ca/fossil-fuel-advertisements
Fossil Free Parliament - UK-wide campaign to free parliament from the influence of fossil fuel industry- https://fossilfreeparliament.uk/

## Slide 14
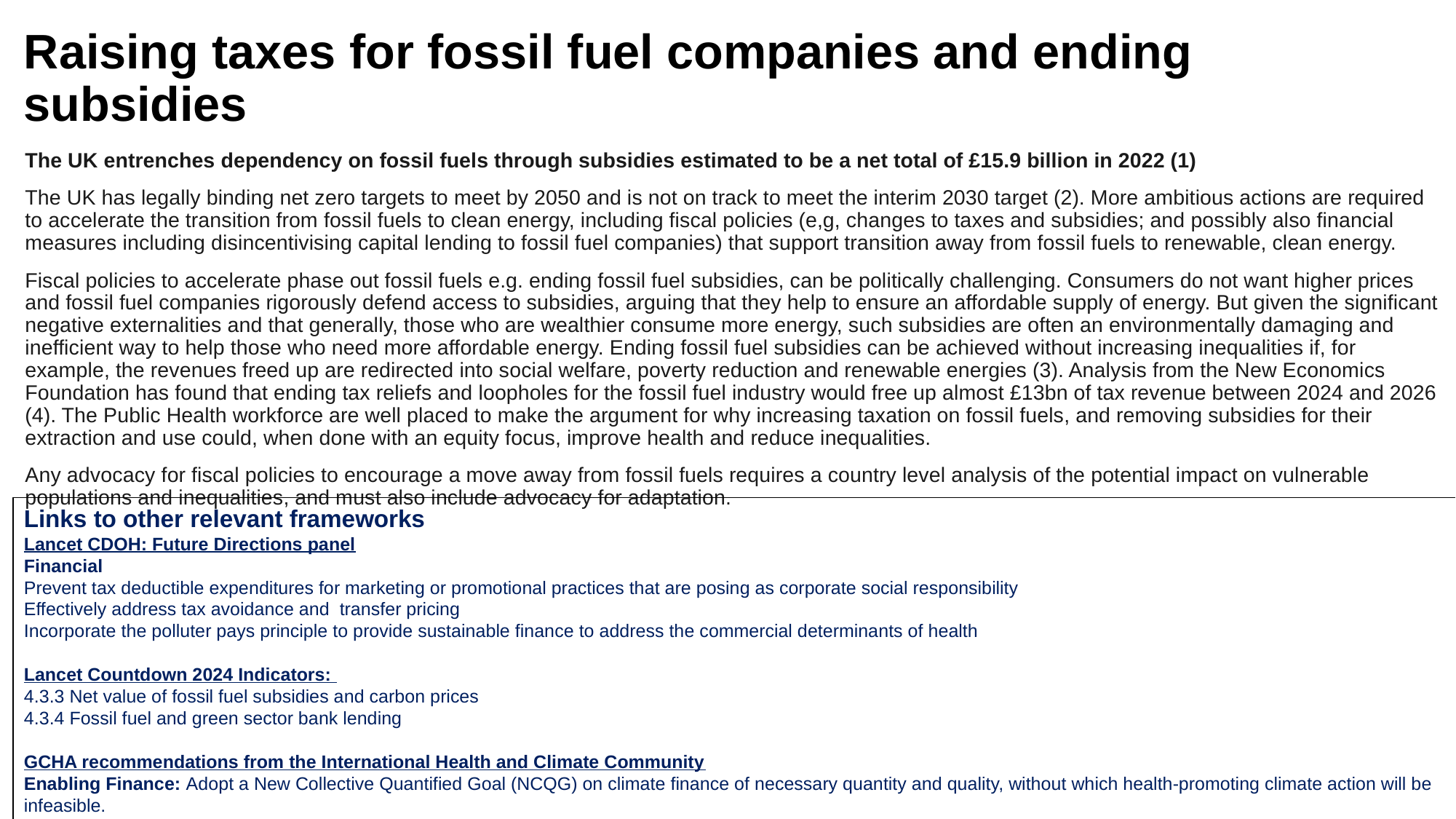

# Raising taxes for fossil fuel companies and ending subsidies
The UK entrenches dependency on fossil fuels through subsidies estimated to be a net total of £15.9 billion in 2022 (1)
The UK has legally binding net zero targets to meet by 2050 and is not on track to meet the interim 2030 target (2). More ambitious actions are required to accelerate the transition from fossil fuels to clean energy, including fiscal policies (e,g, changes to taxes and subsidies; and possibly also financial measures including disincentivising capital lending to fossil fuel companies) that support transition away from fossil fuels to renewable, clean energy.
Fiscal policies to accelerate phase out fossil fuels e.g. ending fossil fuel subsidies, can be politically challenging. Consumers do not want higher prices and fossil fuel companies rigorously defend access to subsidies, arguing that they help to ensure an affordable supply of energy. But given the significant negative externalities and that generally, those who are wealthier consume more energy, such subsidies are often an environmentally damaging and inefficient way to help those who need more affordable energy. Ending fossil fuel subsidies can be achieved without increasing inequalities if, for example, the revenues freed up are redirected into social welfare, poverty reduction and renewable energies (3). Analysis from the New Economics Foundation has found that ending tax reliefs and loopholes for the fossil fuel industry would free up almost £13bn of tax revenue between 2024 and 2026 (4). The Public Health workforce are well placed to make the argument for why increasing taxation on fossil fuels, and removing subsidies for their extraction and use could, when done with an equity focus, improve health and reduce inequalities.
Any advocacy for fiscal policies to encourage a move away from fossil fuels requires a country level analysis of the potential impact on vulnerable populations and inequalities, and must also include advocacy for adaptation.
Links to other relevant frameworks
Lancet CDOH: Future Directions panel
Financial
Prevent tax deductible expenditures for marketing or promotional practices that are posing as corporate social responsibility
Effectively address tax avoidance and transfer pricing
Incorporate the polluter pays principle to provide sustainable finance to address the commercial determinants of health
Lancet Countdown 2024 Indicators:
4.3.3 Net value of fossil fuel subsidies and carbon prices
4.3.4 Fossil fuel and green sector bank lending
GCHA recommendations from the International Health and Climate Community
Enabling Finance: Adopt a New Collective Quantified Goal (NCQG) on climate finance of necessary quantity and quality, without which health-promoting climate action will be infeasible.

## Slide 15
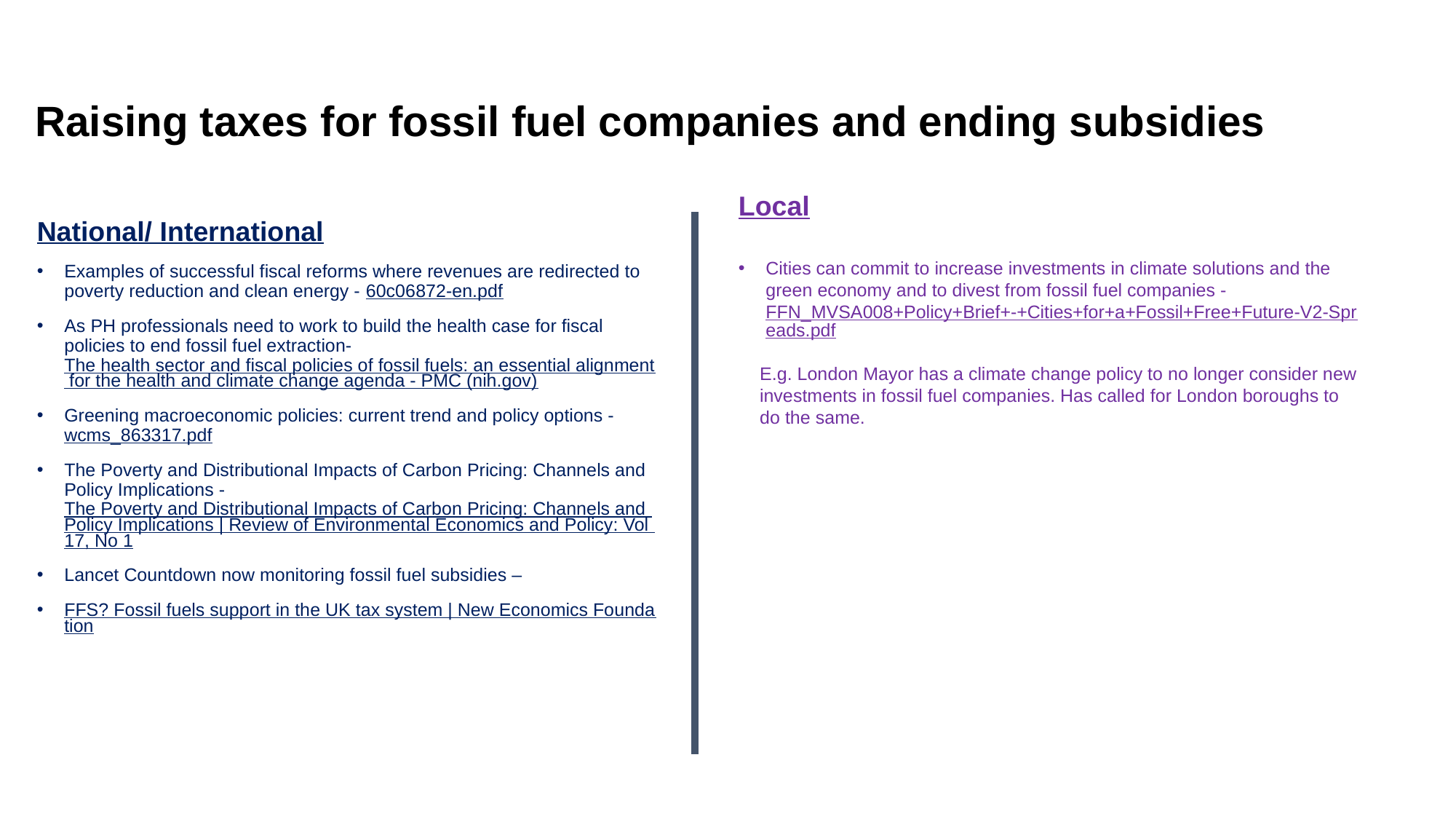

# Raising taxes for fossil fuel companies and ending subsidies
Local
Cities can commit to increase investments in climate solutions and the green economy and to divest from fossil fuel companies - FFN_MVSA008+Policy+Brief+-+Cities+for+a+Fossil+Free+Future-V2-Spreads.pdf
E.g. London Mayor has a climate change policy to no longer consider new investments in fossil fuel companies. Has called for London boroughs to do the same.
National/ International
Examples of successful fiscal reforms where revenues are redirected to poverty reduction and clean energy - 60c06872-en.pdf
As PH professionals need to work to build the health case for fiscal policies to end fossil fuel extraction- The health sector and fiscal policies of fossil fuels: an essential alignment for the health and climate change agenda - PMC (nih.gov)
Greening macroeconomic policies: current trend and policy options - wcms_863317.pdf
The Poverty and Distributional Impacts of Carbon Pricing: Channels and Policy Implications - The Poverty and Distributional Impacts of Carbon Pricing: Channels and Policy Implications | Review of Environmental Economics and Policy: Vol 17, No 1
Lancet Countdown now monitoring fossil fuel subsidies –
FFS? Fossil fuels support in the UK tax system | New Economics Foundation

## Slide 16
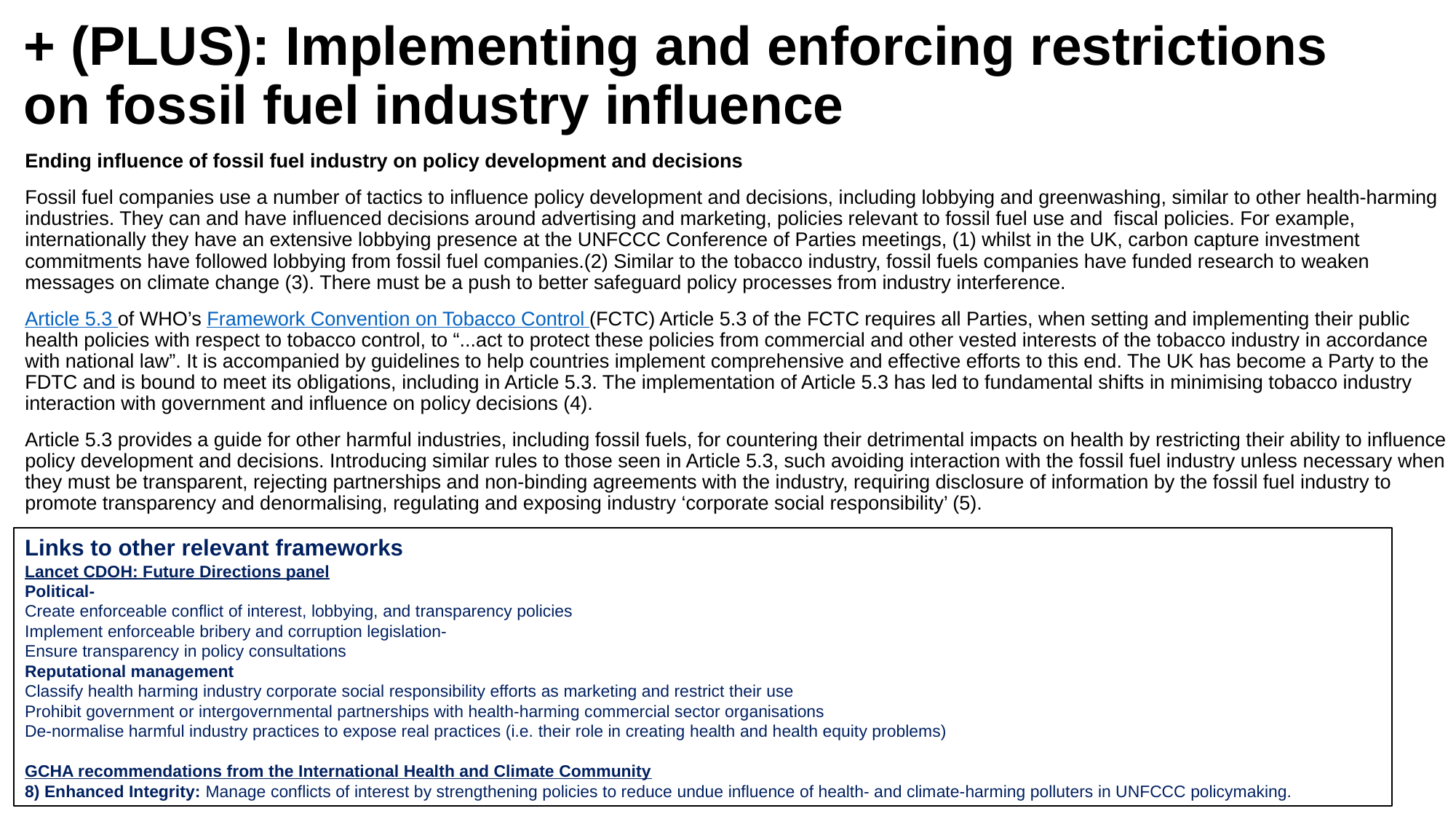

# + (PLUS): Implementing and enforcing restrictions on fossil fuel industry influence
Ending influence of fossil fuel industry on policy development and decisions
Fossil fuel companies use a number of tactics to influence policy development and decisions, including lobbying and greenwashing, similar to other health-harming industries. They can and have influenced decisions around advertising and marketing, policies relevant to fossil fuel use and fiscal policies. For example, internationally they have an extensive lobbying presence at the UNFCCC Conference of Parties meetings, (1) whilst in the UK, carbon capture investment commitments have followed lobbying from fossil fuel companies.(2) Similar to the tobacco industry, fossil fuels companies have funded research to weaken messages on climate change (3). There must be a push to better safeguard policy processes from industry interference.
Article 5.3 of WHO’s Framework Convention on Tobacco Control (FCTC) Article 5.3 of the FCTC requires all Parties, when setting and implementing their public health policies with respect to tobacco control, to “...act to protect these policies from commercial and other vested interests of the tobacco industry in accordance with national law”. It is accompanied by guidelines to help countries implement comprehensive and effective efforts to this end. The UK has become a Party to the FDTC and is bound to meet its obligations, including in Article 5.3. The implementation of Article 5.3 has led to fundamental shifts in minimising tobacco industry interaction with government and influence on policy decisions (4).
Article 5.3 provides a guide for other harmful industries, including fossil fuels, for countering their detrimental impacts on health by restricting their ability to influence policy development and decisions. Introducing similar rules to those seen in Article 5.3, such avoiding interaction with the fossil fuel industry unless necessary when they must be transparent, rejecting partnerships and non-binding agreements with the industry, requiring disclosure of information by the fossil fuel industry to promote transparency and denormalising, regulating and exposing industry ‘corporate social responsibility’ (5).
Links to other relevant frameworks
Lancet CDOH: Future Directions panel
Political-
Create enforceable conflict of interest, lobbying, and transparency policies
Implement enforceable bribery and corruption legislation-
Ensure transparency in policy consultations
Reputational management
Classify health harming industry corporate social responsibility efforts as marketing and restrict their use
Prohibit government or intergovernmental partnerships with health-harming commercial sector organisations
De-normalise harmful industry practices to expose real practices (i.e. their role in creating health and health equity problems)
GCHA recommendations from the International Health and Climate Community
8) Enhanced Integrity: Manage conflicts of interest by strengthening policies to reduce undue influence of health- and climate-harming polluters in UNFCCC policymaking.

## Slide 17
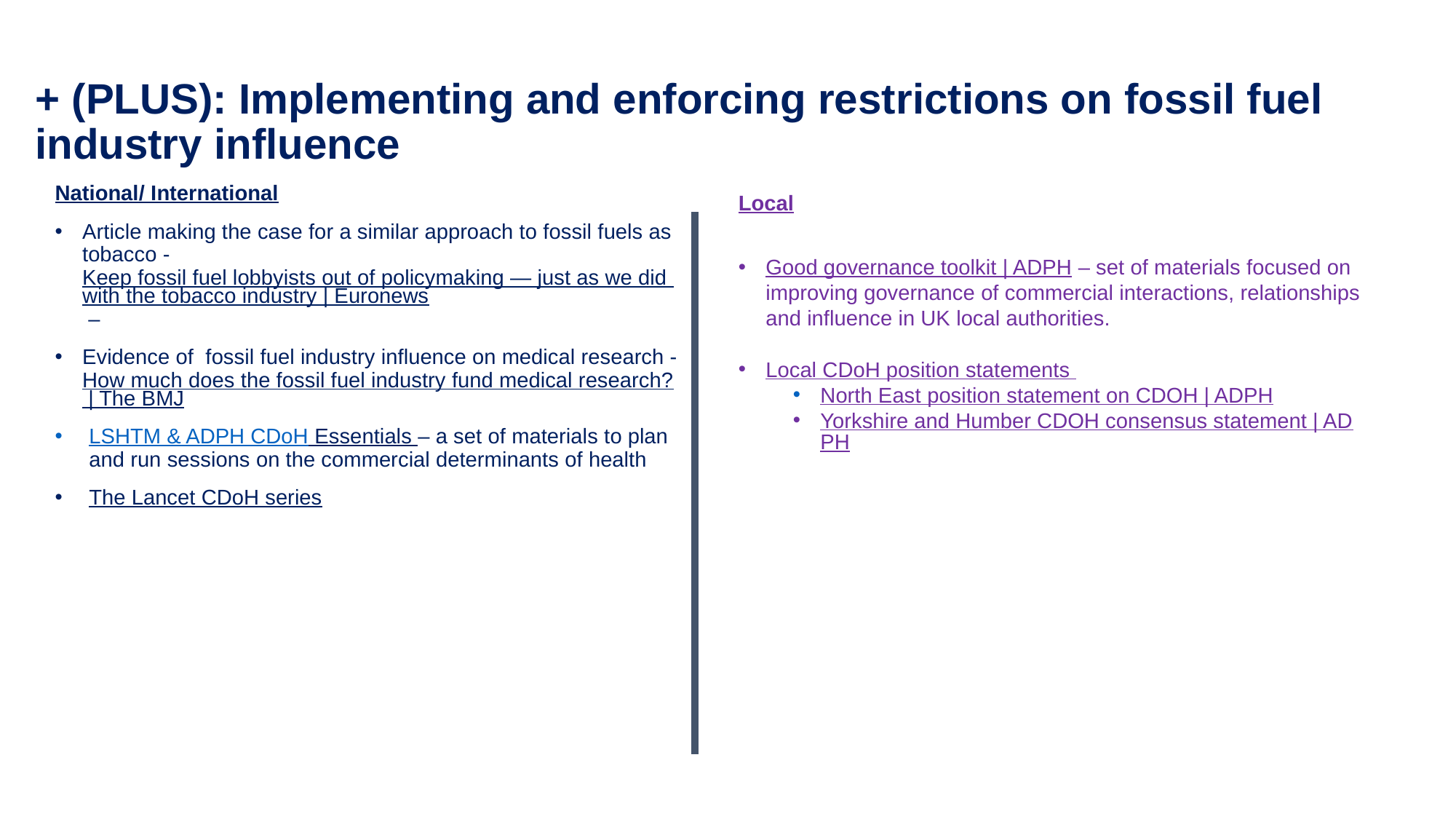

# + (PLUS): Implementing and enforcing restrictions on fossil fuel industry influence
National/ International
Article making the case for a similar approach to fossil fuels as tobacco - Keep fossil fuel lobbyists out of policymaking — just as we did with the tobacco industry | Euronews –
Evidence of fossil fuel industry influence on medical research - How much does the fossil fuel industry fund medical research? | The BMJ
LSHTM & ADPH CDoH Essentials – a set of materials to plan and run sessions on the commercial determinants of health
The Lancet CDoH series
Local
Good governance toolkit | ADPH – set of materials focused on improving governance of commercial interactions, relationships and influence in UK local authorities.
Local CDoH position statements
North East position statement on CDOH | ADPH
Yorkshire and Humber CDOH consensus statement | ADPH

## Slide 18
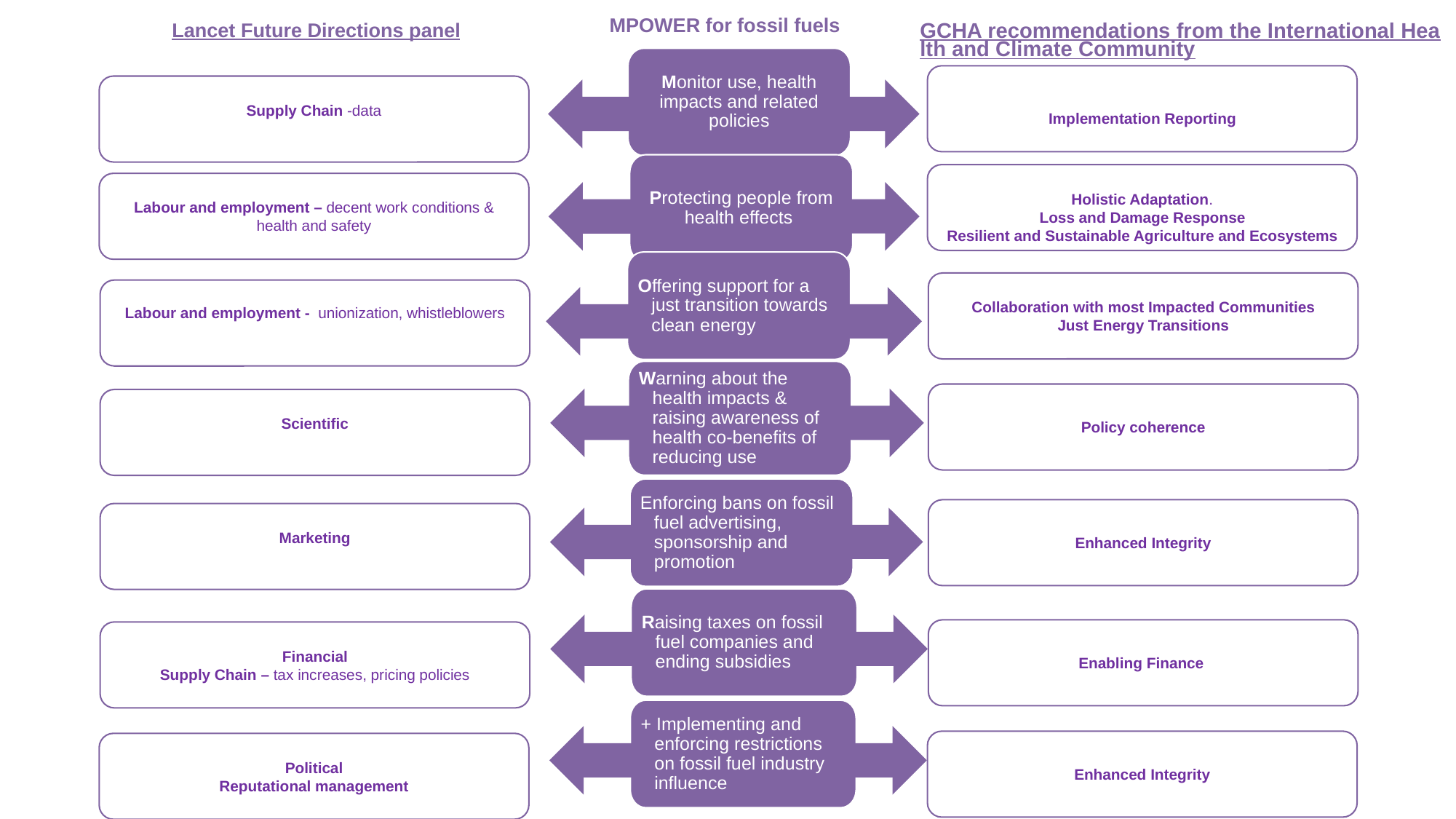

GCHA recommendations from the International Health and Climate Community
MPOWER for fossil fuels
Lancet Future Directions panel
Monitor use, health impacts and related policies
Implementation Reporting
Supply Chain -data
Protecting people from health effects
Holistic Adaptation.
Loss and Damage Response
Resilient and Sustainable Agriculture and Ecosystems
Labour and employment – decent work conditions & health and safety
Offering support for a just transition towards clean energy
Collaboration with most Impacted Communities
Just Energy Transitions
Labour and employment - unionization, whistleblowers
Warning about the health impacts & raising awareness of health co-benefits of reducing use
Policy coherence
Scientific
Enforcing bans on fossil fuel advertising, sponsorship and promotion
Enhanced Integrity
Marketing
Raising taxes on fossil fuel companies and ending subsidies
Enabling Finance
Financial
Supply Chain – tax increases, pricing policies
+ Implementing and enforcing restrictions on fossil fuel industry influence
Enhanced Integrity
Political
Reputational management
